# Ocular Speech Tracking Persists in Blindness, but Its Dynamics and Oculo-Cerebral Connectivity Depend on Visual Status

**DOI:** 10.1523/ENEURO.0041-26.2026

**Published:** 2026-07-07

**Authors:** Kaja Rosa Benz, Larissa Reitinger, Fabian Schmidt, Davide Bottari, Anne Hauswald, Olivier Collignon, Nathan Weisz

**Affiliations:** ^1^Centre for Cognitive Neuroscience, Department of Psychology, Paris-Lodron-University of Salzburg, Salzburg 5020, Austria; ^2^Basque Center on Cognition, Brain and Language, Donostia - San Sebastián 20009, Spain; ^3^IMT School for Advanced Studies Lucca, Lucca 55100, Italy; ^4^Center for Mind/Brain Sciences, University of Trento, Rovereto 38068, Italy; ^5^Institute of Neuroscience (IoNS), UCLouvain, Louvain-la-Neuve B-1348, Belgium; ^6^Neuroscience Institute, Christian Doppler University Hospital, Paracelsus Medical University Salzburg, Salzburg 5020, Austria

**Keywords:** blind, eye movements, speech tracking, vocoding

## Abstract

While eye movements have been shown to track the speech envelope, it is unknown whether this reflects a hard-wired mechanism or one shaped by (lifetime) audiovisual experience. Further, questions remain about whether ocular tracking is modulated by speech intelligibility and which brain regions drive these synchronized eye movements. Here, we investigate ocular speech tracking in 47 (20 male), blindfolded early blind, late blind, and sighted individuals using magnetoencephalography and source-reconstructed oculomotor signals while participants listened to narrative speech of varying intelligibility. We find that oculomotor activity tracks acoustic speech features; however, while neural speech tracking is modulated by intelligibility, ocular tracking patterns remain ambiguous. Interestingly, we find effects reflected in two frequency-specific components: a low-frequency (∼1 Hz) effect present across all groups, indicating that visual experience is not required, and a high-frequency (∼6 Hz) effect reduced in early and late blind individuals. Moreover, this finding is not driven by cerebro-ocular connectivity, as late blind individuals exhibit stronger connectivity between the eyes and the left temporal cortices without a corresponding increase in ocular tracking. In conclusion, ocular speech tracking seems to respond selectively to acoustic features of speech, and does not require visual experience to develop. It may thus represent a hard-wired oculomotor mechanism within the oculo-cerebral network involved in speech processing.

## Significance Statement

Eye movements provide a unique window into the interaction between auditory and visual systems. By studying early blind, late blind, and sighted individuals, we demonstrate that speech-related eye movements arise from at least two distinct mechanisms: a low-frequency component that occurs independently of (lifetime) visual experience and is linked to processing of acoustic speech features, and a high-frequency component shaped by prior visual exposure. Importantly, while neural measures were clearly modulated by speech intelligibility, the corresponding ocular responses yielded statistically ambiguous patterns. This potential dissociation suggests that eye movements may reflect mechanisms of spoken language processing that operate independently of intelligibility, revealing novel pathways of auditory-motor coupling that warrant further causal investigation.

## Introduction

In most settings, speech perception is an audiovisual process, drawing on a hierarchy of processing stages to support comprehension (Keough et al., 2019; Hamilton et al., 2021; Banks et al., 2023; Reisinger et al., 2023). These processes range from sensory encoding of acoustic speech features to higher-level integration of linguistic features (Hickok and Poeppel, 2007; Herbet and Duffau, 2020). Efficient processing relies on neural systems dynamically tracking these features (Peelle and Davis, 2012; Doelling et al., 2014), known as neural speech tracking (Obleser and Kayser, 2019). Recent findings demonstrate that speech is also tracked by eye movements. Informed by a substantial body of research on audiovisual integration highlighting the involvement of eye movements in speech processing (Porter et al., 2007; Bulkin and Groh, 2012; Lee and Groh, 2012; Jin et al., 2018; Leszczynski et al., 2023; Gehmacher et al., 2024a) established that eye movements track the speech envelope of continuous, narrative, attended speech—a phenomenon termed ocular speech tracking. Ocular tracking was strongest for the attended speaker in multi-speaker settings, and higher tracking was observed in participants reporting lower comprehension (Gehmacher et al., 2024a; Schubert et al., 2024), suggesting a compensatory attentional role under demanding listening conditions (Schubert et al., 2024). Listening effort is often probed by degrading intelligibility using vocoded speech, which reduces spectral detail while preserving the temporal envelope (Shannon et al., 1995). Our previous work shows that neural speech tracking modulates as a function of speech intelligibility (Hauswald et al., 2022; Chen et al., 2023; Schmidt et al., 2023), but whether ocular tracking shows a similar modulation or reflects sensitivity to acoustic dynamics remains unknown. A further central open question is whether ocular speech tracking requires concurrent retinal input. Previous work tested only sighted participants with open eyes but no task-relevant visual input, leaving unresolved whether ocular tracking persists without visual input. Another question concerns whether ocular tracking develops in the absence of (lifetime) visual experience. If the development of ocular tracking requires visual experience, early blind individuals should lack it; if it reflects a hard-wired mechanism, it should occur independently of visual status. We previously showed that during silent lip-reading, auditory deprivation is influencing ocular tracking of the (unheard) speech envelope, as late deafs exhibit increased ocular tracking while early deafs did not show envelope tracking (Benz et al., 2025). Although controlling for eye movements did not account for the observed neural effects in that work, in auditory speech, bidirectional interactions between oculomotor and neural systems (Gehmacher et al., 2024a; Madsen and Parra, 2024) appear to play an important role in ocular speech tracking. Establishing how ocular activity couples with the brain during speech perception may clarify whether eye movements are driven by top-down cortical control or provide bottom-up input to auditory processing in the absence of visual input. Early blind individuals retain functional circuitry between motor cortex and oculomotor activity (Koba et al., 2024). In line with this, enhanced hearing abilities in this population in comparison to sighted individuals have been reported and linked to activation in the visual cortex (Sabourin et al., 2022), which is also engaged in spoken word processing (Matuszewski et al., 2025). This leads to the hypothesis that, in the absence of vision, eye movements may still support auditory processing. However, findings of reduced eye-visual cortex connectivity in early blindness (Koba et al., 2024) challenge this idea. Alternatively, it is possible that the eyes of blind individuals are still involved in speech perception but relay information directly to the ears (King et al., 2023; Lovich et al., 2023), without involving the visual cortex. Since these eye-to-ear connections do not rely on visual input (Abbasi et al., 2025), and blind participants exhibit eye movement patterns comparable to those of sighted individuals (Händel et al., 2025), ocular speech tracking might occur independently of being blind or not. Here, we directly address these open questions by reanalyzing magnetoencephalography (MEG) data from a previously published dataset (early blind, *n* = 17; sighted, *n* = 14; Van Ackeren et al., 2018) extended by a late blind group (*n* = 16). Participants were blindfolded and listened to continuous, narrative speech presented at three intelligibility levels (natural, 8-channel vocoded, and 1-channel vocoded). We extracted eye movement time-series data using source reconstruction and computed coherence to investigate ocular and neural speech tracking and functional oculo-cerebral connectivity. Specifically, we asked: (1) Does ocular speech tracking occur while participants listen to speech in the absence of concurrent visual input, and is it modulated by intelligibility? (2) Does its presence and strength depend on lifetime visual experience? (3) Which neural systems drive ocular tracking, and how does oculo-cerebral connectivity vary with visual status? By addressing these questions, we test whether ocular speech tracking reflects a hard-wired, experience-independent mechanism or one shaped by visual experience, and investigate how it relates to established neural speech tracking processes.

## Materials and Methods

### Participants

Seventeen early blind (7 male; 33.6 ± 10.55 years; 20–67 years), 16 late blind (9 male; 45.5 ± 16.62 years; 26–71 years), and 14 sighted participants (4 male; 33.2 ± 13.06 years; 20–63 years) participated in the present study. Groups were matched in terms of age and biological sex (Van Ackeren et al., 2018). All participants were proficient braille readers and native Italian speakers. None of them suffered from a known neurological or peripheral auditory disease. All early blind participants were either totally blind or severely visually impaired from birth; however, two reported residual visual perception before the age of 3, one before the age of 4, and one participant lost their sight completely at age 10. Residual visual perception of diffuse light was present in 8 early blind participants. Causes of vision loss included retinal damage or detachment (10), optic nerve damage (3), infection of the eyes (1), microphthalmia (2), and hypoxia (1). In the late blind group, the age of blindness onset varied from childhood to adulthood. Importantly, they were all born with intact vision, which was not the case for the early blind. Residual visual perception of diffuse light was present in 10 late blind participants, and 5 had no residual vision. Causes of late blindness included retinopathy (7), congenital glaucoma (3), damaged optic nerve (4), eye injury (1), and congenital cataracts (1). Participants signed informed consent forms before participation. The project was approved by the Ethics Committee of the University of Trento and was in accordance with the Declaration of Helsinki.

### Experimental design

The present study builds on data from Van Ackeren et al. (2018). Auditory stimuli were presented within the magnetically shielded MEG room via stereo loudspeakers employing a Panphonics Sound Shower Two amplifier, calibrated to a consistent and comfortable volume level across all participants. The presentation of the stimulus was controlled using Psychophysics Toolbox 3 (http://psychtoolbox.org; RRID:SCR002881) running in MATLAB (RRID:SCR001622), executed on a Dell Alienware Aurora workstation operating on 64-bit Windows 7. To eliminate visual input, both sighted and blind individuals were blindfolded throughout the experimental session. The room was dimly illuminated to permit video-based observation of participants during MEG acquisition. Experimental instructions were provided via pre-recorded audio messages spoken by one of the experimenters. The speakers mean syllable rate was 5.99 Hz (std: 3.01 Hz; calculated with syllable nuclei [De Jong and Wempe, 2009] computed using the Python library parselmouth [Jadoul et al., 2018]). The stimulus set consisted of 14 one-minute excerpts derived from well-known Italian audiobooks (i.e., Pippi Longstocking and Candide). Two additional conditions were generated through channel vocoding using Praat software (Boersma, 2007). Specifically, the original audio signal was decomposed into either one (1-channel) or eight (8-channel) logarithmically spaced frequency bands. For each band, the amplitude envelope was extracted and used to amplitude-modulate band-limited Gaussian noise (noise carrier). The modulated noise bands were then recombined into a single audio signal, preserving the overall temporal envelope while progressively degrading spectral detail. This manipulation preserved the overall amplitude envelope while gradually degrading spectral detail. In perceptual terms, the 8-channel version produced substantial vocal distortion without impairing intelligibility, whereas the 1-channel version rendered speech entirely unintelligible. Thus, the 1-channel vocoded condition represents the most degraded and least intelligible stimulus. In total, 42 stimuli were presented across seven pseudo-randomized blocks. Each block included two exemplars from each condition, with no repetition of narrative content within a block. To assess comprehension, each stimulus segment was followed by a single declarative sentence summarizing the story content. Participants were instructed to attend closely to each passage and to indicate whether the statement was true or false using a non-magnetic response box, operated with the index and middle fingers of the right hand.

### MEG data recording

The experiment was conducted in the MEG Lab at the Center for Mind/Brain Sciences (Laboratorio di magnetoencefalografia at CiMeC) in Rovereto in Italy. Whole-head MEG was acquired using a triple-sensor (204 planar gradiometers; 102 magnetometers) 306-channel system (Elekta Neuromag). Continuous recordings had a sampling rate of 1,000 Hz and were band-pass filtered online between 0.1 and 300 Hz. Individual head shapes were digitized with a Polhemus FASTRAK 3D tracking system (Polhemus). Five localization coils were used to continuously record the head position of the subject.

### MEG data preprocessing

**Figure 1.**
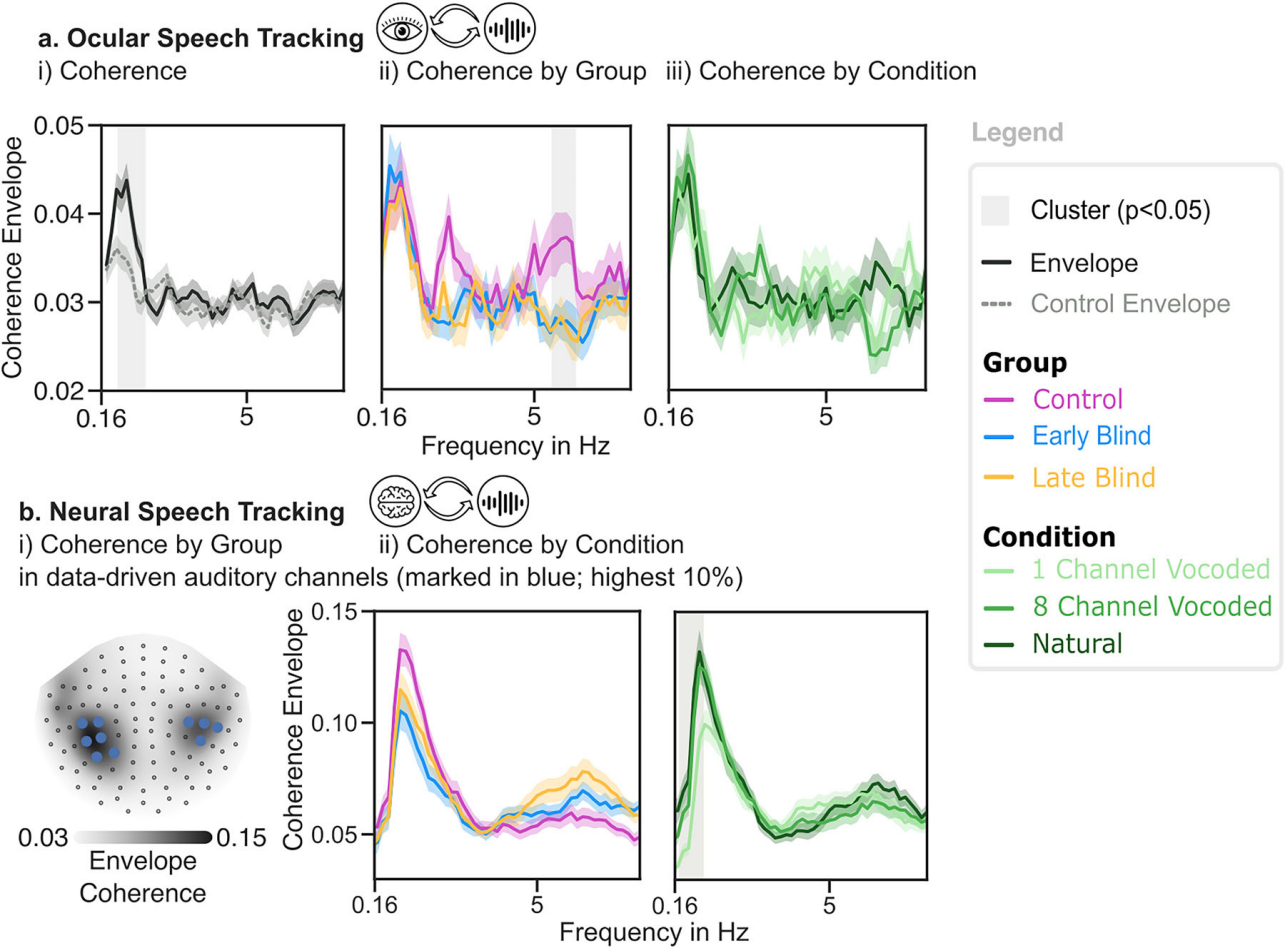
***a***, Ocular response to speech is (i) present with eyes closed (ii) increased in sighted individuals from 5.5 to 6.33 Hz compared to the early and late blind individuals (iii) not significantly modulated by intelligibility; however, equivalence testing failed to confirm statistical equivalence. ***b***, (i) Channels with the maximal envelope coherence at 1 Hz were selected as auditory channels, (ii) visualization of the main effects in the auditory channels reveal different patterns than the ocular ones. For source reconstruction sanity checks and mTRF results (Figure 1-1) and for ICA eye components (Figure 1-2).

**Figure 2,:**
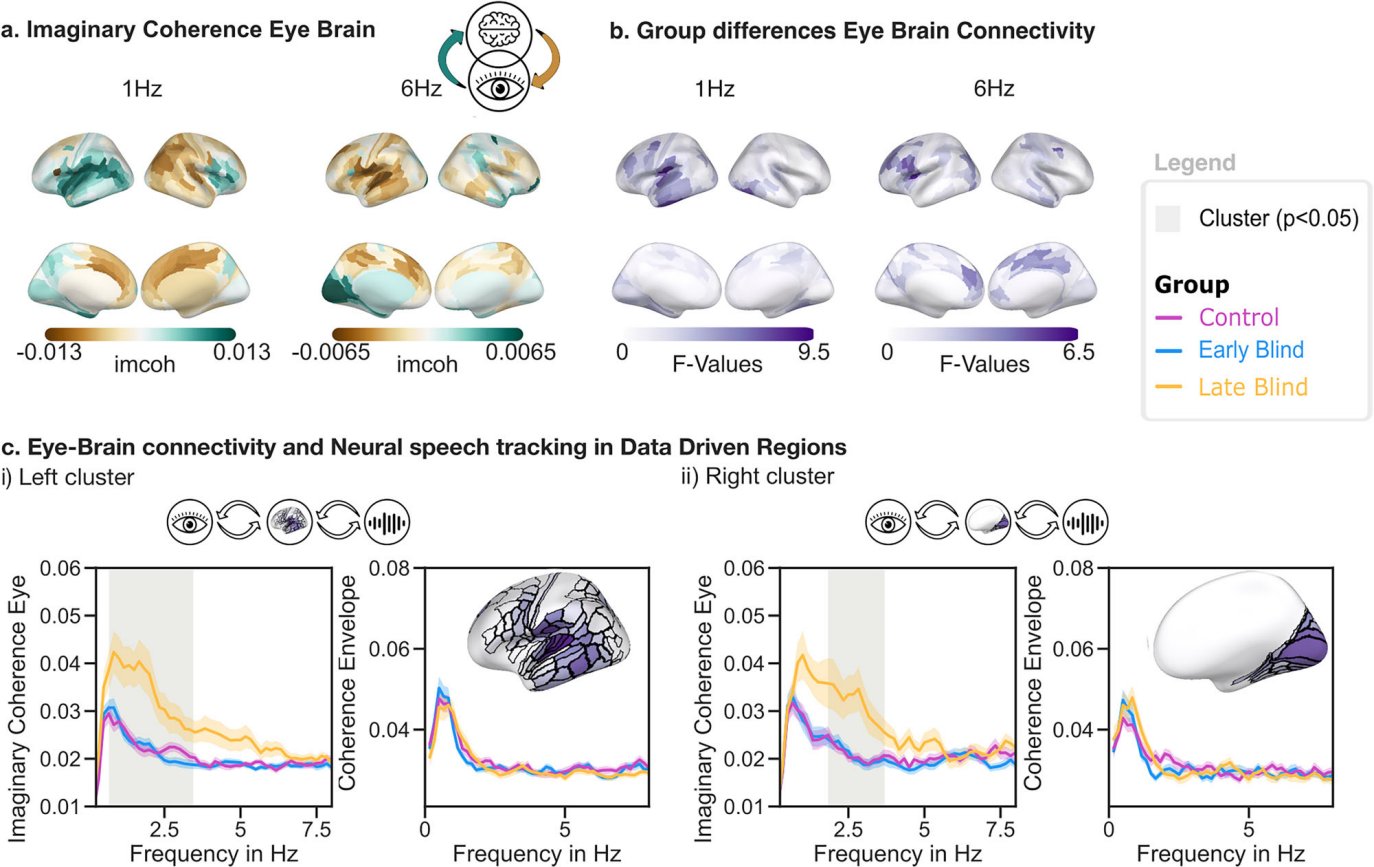
***a***, Imaginary coherence without any masking. The imaginary part of coherence indicates oculo-cerebral connectivity when positive (green) and cerebro-ocular connectivity when negative (brown). ***b***, Group comparison of oculo-cerebral connectivity between the three groups different visual status: uncorrected *F*-values of the absolute value of the imaginary coherence. ***c***, Brain areas extracted from the cluster-based permutation test of the absolute value of the imaginary coherence. These plots reveal significantly increased oculo-cerebral imaginary coherence in the late blind group. A list of the HCPMMP1 regions included in the clusters can be found in Figure 2-1 (right hemisphere) and Figure 2-2 (left hemisphere). Follow-up analyses of cerebro-acoustic coherence in these regions showed no group differences.

Data preprocessing was performed in MNE-Python (Gramfort, 2013). Initially, all recordings were processed with a signal space separation algorithm (Maxwell filter; Taulu and Kajola, 2005) that removes external magnetic interference and corrects for within-session head movement. For this, the data from each individual participant were realigned to their mean head position across blocks. For independent component analysis (fast ICA; Hyvarinen, 1999), a 1 Hz high-pass filter was applied to the raw data and it was downsampled to 150 Hz, yielding 50 components. Cardiac artifacts were identified automatically with the default function in MNE-Python. The components were applied to the original raw data, and the cardiac artifact removed. This ICA-corrected data was filtered between 0.1 and 16 Hz using a Blackman filter and the default MNE raw filter settings and also downsampled to 150 Hz. We subsequently attempted to identify consistent ocular-related ICA components using semi-automated methods, including correlation with frontal channel activity, template matching (Campos Viola et al., 2009), and evaluation of component topography symmetry. However, although we were able to extract good components (extended data, Figs. 1-1, 1-2), we were not successful in consistently identifying the symmetric topography we expected across groups (no convincing symmetric component for 17% of the participants), likely because participants were blindfolded with a heavy bandage to avoid voluntary eye movements and blinks. Given the inadequacy of ICA to model ocular activity consistently in this particular study, we implemented a source reconstruction approach for this purpose. Note that for the sensor plots and statistics we used the magnetometers only, but for the source reconstruction, all channels were included.

#### Source projection of MEG data

We employed a spatial filtering approach (LCMV beamformer) to reconstruct both ocular and neural sources. Source projection of the data was performed with MNE-Python (Gramfort, 2013). An automatic coregistration pipeline was used to align the FreeSurfer “fsaverage” template brain (Fischl, 2012) to each participant’s head shape. After an initial fit using the three fiducial landmarks, the coregistration was refined with the iterative closest point algorithm (Besl and McKay, 1992). Head shape points located more than 5 mm away from the scalp were automatically omitted. Since a single-layer boundary element model (BEM; Akalin-Acar and Gençer, 2004) does not include the eyes, a sphere model was used as the BEM parameter in the present study. Next, a surface source space was defined using the ico-4 subdivision (5,124 sources in total, 2,562 per hemisphere), and the eyeballs were added as a volume source space with 514 sources. Based on the fsaverage template, the eyes were modeled as two spheres positioned at the locations of the eyes in the template MRI. The forward operator (i.e., lead field matrix) was then computed using the individual coregistration, the sphere model, and the mixed source space (surface for the brain and volume for the eyes). For further analysis, the principal component of each region from a multi-modal parcellation of the human cerebral cortex (HCPMMP1; Glasser et al., 2016) was used for visualization and statistical analyses. Consistent with the approach for brain regions, the first principal component from the principal component analysis of the eye-source reconstructions was used for further analysis. We want to emphasize, that the distinct spatial location of the eyes allows them to be modeled as independent, likely even more reliably than a single brain area with less spatial separation.

#### Speech envelope extraction

Amplitude envelopes of the auditory stimuli were derived using the Chimera toolbox (Chandrasekaran et al., 2009) in combination with custom scripts, following the methodology outlined by Gross et al. (2013). First, the audio files were band-pass filtered into nine frequency bands from 100 to 1,000 Hz using a fourth-order Butterworth filter. To avoid phase shifts relative to the original signal, filtering was applied in both forward and backward directions. Frequency bands were evenly spaced to correspond with the human basilar membrane’s tonotopic organization. The analytic amplitude for each filtered segment was computed as the absolute of the Hilbert transform. Subsequently, the amplitude envelopes across all bands were summed and normalized to a maximum value of one. This composite envelope was then also downsampled to 150 Hz and temporally aligned with the MEG recordings and processed using identical analysis pipelines. For the control envelope, the envelope of the respective audio file was cut into two pieces and the two parts were exchanged.

### Speech tracking

To investigate neural speech tracking, two complementary analytical approaches are commonly used: frequency-domain measures, such as coherence, and regression-based methods, such as the multivariate temporal response function (mTRF; Lalor and Nidiffer, 2025). Both approaches have also previously been applied to investigate ocular speech tracking (Gehmacher et al., 2024a; Schubert et al., 2024; Benz et al., 2025). To obtain an integrated picture with both methods, we decided to utilize and present both approaches with coherence and mTRFs. As demonstrated in previous studies, the results have yielded either consistent or complimentary results across varying levels of intelligibility (Chen et al., 2023) and diverse linguistic backgrounds and exposure (Pérez-Navarro et al., 2023). The coherence results are presented in the main body of the manuscript, and the (mainly consistent) mTRF results are illustrated in the extended data (Fig. 1-1).

#### Coherence

For the coherence analysis, the data were segmented into epochs of 6 s to ensure sufficient resolution of the low-frequency oscillations. A constant detrending procedure was applied to the epochs, thereby removing any offset without altering the linear trend. In addition, baseline correction was applied using the interval from 0 to 0.5 s, ensuring that subsequent analyses were referenced to this period. Coherence was then calculated using the spectral-connectivity-epochs function from MNE-Python, applied to the epoched data. We used multitaper spectral estimation, without adaptive weighting, and without frequency averaging, to preserve spectral resolution. Coherence was computed across the frequency range between 0.1 and 8 Hz to include delta (<4 Hz) and theta (4–8 Hz) bands. The resulting measures are cerebro-acoustic coherence as a measure for neural tracking, and ocular-acoustic coherence as a measure for ocular tracking. To test the connectivity between the eyes and the brain, we used the imaginary part of coherence, in order to avoid volume conduction effects (Nolte et al., 2004). All other settings were identical to the coherence calculation. Imaginary coherence yields positive and negative values, with the sign indicative of the directionality of connectivity (here: positive indicates oculo-cerebral [i.e., eye-to-brain] connectivity, and negative cerebro-ocular [i.e., brain-to-eye] connectivity). These were analyzed in a descriptive manner, and, we advise caution regarding the interpretation of directionality. Imaginary connectivity is a circular measure that we used to show both the negative and positive phase lag. At 6 Hz, the 166 ms cycle length is shorter than the 250 ms ocular response time reported by Gehmacher et al. (2024b). This introduces potential phase-wrapping issues, as a response occurring at 250 ms would exceed a full cycle, making it mathematically difficult to distinguish a true lead from a lag. Nevertheless, as the effect remains robust across the group average and is highly compelling, we have chosen to report these findings here. For statistics, we used the absolute value of the imaginary coherence.

#### Multivariate temporal response function

For the estimation of mTRFs, we used Eelbrain’s boosting implementation (Brodbeck et al., 2023) of the full time-series data. For each of the three conditions, we concatenated all blocks, resulting in 8 min of data per participant, on which the boosting analysis was ran. As Bialas and Lalor (2025) suggest 5–15 s length of the partition, the data was partitioned into 50 folds (leading to around 10 s per fold), with one fold used as the test set in each iteration. The model was fit for a temporal window from −0.4 to 0.8 s relative to stimulus time-series (Schubert et al., 2024). We used an l1 error metric to promote sparsity in the model. The mTRFs were estimated using a basis function width of 50 ms, allowing temporal smoothing of the response functions. To avoid overfitting, selective stopping was enabled, so that boosting was halted independently for each predictor when it no longer improved model performance on the validation data.

#### Statistics

To test for differences in frequency bands, time-series, and sensors, we ran a cluster-based permutation test to correct for multiple comparisons in eelbrain (Brodbeck et al., 2023). For this, the whole range was used: all magnetometers, full frequency range for the coherence (0.25 to 8 Hz), and a time range of interest for the mTRF (−100 to 800 ms). For the sensor plots in 1b) we pre-selected the sensors with the highest 10% of envelope coherence at 1 Hz, and called them auditory sensors in order to make sure that we are not confusing auditory activity with the ocular activity data. When comparing for the control versus the actual envelope, a cluster-based permutation *t*-test was conducted. For comparing the groups and conditions, a mixed-design ANOVA with the dependent variable ocular tracking (mTRF or coherence, respectively) and factors group (between-subjects) and condition (within-subjects) was run. For the source-reconstructed data, we conducted a cluster-based permutation test with controlling for neighboring regions. We plot the uncorrected *F*-values in Figure 2*b*, thereby enabling interpretation of the spatial patterns at 1 and 6 Hz, and show the resulting clusters of the cluster-based permutation test in Figure 2*c*. For post hoc analysis of the ANOVA, paired *t*-tests with Bonferroni correction were conducted. To formally test for the absence of an effect regarding intelligibility and group status on 1 Hz ocular coherence, we performed the two one-sided tests (TOST) procedure. Both tests were conducted in Python with Pingouin (Vallat, 2018). All statistical analyses were performed on subject-level data, with each participant contributing one observation per condition to the respective tests.

## Results

In the present study, we investigated ocular and neural speech tracking in early blind, late blind, and sighted individuals while they blindfoldedly listened to continuous, narrative speech presented at three different intelligibility levels (natural, 1-channel vocoded, and 8-channel vocoded) without relying on direct eye-tracking methods. Instead, we used source reconstruction to estimate ocular activity. Additionally, we investigated the connectivity between ocular and neural sources depending on visual status. Our primary aim was to assess whether (lifetime) visual input is needed for ocular speech tracking to occur, to investigate whether ocular tracking modulates with speech intelligibility, and to depict which neural systems drive ocular tracking.

### Ocular activity during eyes closed and blindfolded listening tracks speech envelope

In a first step, we show across all groups that the reconstructed eye movements track speech acoustics, even when listening with closed and blindfolded eyes. This means that oculomotor activity tracks speech without depending on changing retinal input elicited by eye movements. We compared tracking of the acoustic envelope of the presented audios to a control envelope and found higher tracking of the actually presented acoustic envelope. This was the case for coherence in low frequencies from 0.5 to 1.5 Hz (*t*(47) = 50.34, *p* < 0.005, Fig. 1*a*.i) and for the reconstruction accuracy (*t*(47) = 4.27, *p* < 0.001, 1 − *β* = .99, Fig. 1-1ia). For the mTRF weights, three significant time windows were identified: (1) 220–273 ms (*t*(47) = 85.87, *p* < 0.001), (2) 280–487 ms (*t*(47) = 280.92, *p* < 0.001), and (3) 500–800 ms (*t*(47) = 341.15, *p* < 0.001, Fig. 1-1 i.b). The comparison against a control envelope was used as a validation step to establish stimulus-specific coupling, whereas within- and between-group statistics were focusing on differences in tracking of the presented speech envelope.

10.1523/ENEURO.0041-26.2026.f1-1 Figure 1-1 Ocular and neural speech tracking mTRF analyses and source reconstruction sanity checks. i. Ocular tracking mTRF results. a) Reconstruction accuracy (mean ± SE) of the mTRF model for the speech envelope, compared against a control envelope. Significant tracking is observed for the true envelope (***$p < 0.001$). b) Average temporal response function (TRF weights) across participants. Shaded areas indicate ± SE. Note that the sign of the mTRF weights is not interpretable, as the polarity of the underlying signals is arbitrary. c) Reconstruction accuracy across groups (sighted controls, early blind, late blind) and conditions. A mixed-design ANOVA with the dependent variable Ocular Tracking and factors Group (between-subjects) and Condition (within-subjects) revealed no significant main effect of group ($F(2, 44) = 0.27$, $p = .765$, $\eta^2_g = 0.006$), no significant main effect of condition ($F(44) = 0.73$, $p = .485$, $\eta^2_g = 0.009$), and no significant interaction between group and condition ($F(4, 88) = 0.93$, $p = .448$, $\eta^2_g = 0.022$). ii. To show maximal differences between groups and conditions, and to make sure that the ocular effects are not based on neural speech tracking effects, we plot the sensors with uncorrected $p < 0.01$ (blue) in a time or frequency resolution. Caution: Plots do not do not represent cluster-corrected statistical effect. a) Coherence between selected sensors and the speech envelope at 1 Hz. b) Condition differences in mTRF weights for the selected sensors. c) Group differences in coherence spectra. d) Group differences in mTRF weights. iii. Source reconstruction sanity checks. a) Source-level coherence with the speech envelope at 1 Hz. b) Source-level mTRF reconstruction accuracy. iv. Source reconstructed ocular signal sanity checks a) Coherence between reconstructed ocular activity and neural data at $\sim $1 Hz. This is plotted for both source reconstructed neural data and raw sensor data. The orange sensors reflect the top 5\% of values that are also plotted for the whole frequency range. b) The same procedure has been applied to the mTRF results. The topoplot reflects the reconstruction accuracy of the source reconstructed ocular signal and the sensors. Download Figure 1-1, TIF file.

10.1523/ENEURO.0041-26.2026.f1-2 Figure 1-2 Representative ocular-related ICA components and corresponding time courses across groups. Example independent components classified as ocular-related are shown for five participants per group (early blind, late blind, sighted controls), together with 30-second segments of their time courses. Note that the between-subject variability within the blind groups was higher than in then sighted group, which was one reason for why we opted for source reconstruction. See Methods/MEG Data Preprocessing section for information on ICA-eye channel selection. Download Figure 1-2, TIF file.

10.1523/ENEURO.0041-26.2026.f2-1 Figure 2-1 Group difference oculo-cerebro connectivity. In Fig.2.c.ii, we show the group difference of oculo-cerebro connectivity. Here we list the HCPMMP1 labels of the regions included in clusters in the right hemisphere. The regions are ordered by their contribution to the cluster statistics. The cluster in the right hemisphere spans frequencies from 2 to 3.66 Hz ($F(2,44)=608.07$, $p<0.01$) and peaks in occipital cortex, specifically in V7 (R-V7-ROI-rh). Download Figure 2-1, TIF file.

10.1523/ENEURO.0041-26.2026.f2-2 Figure 2-2 Group difference oculo-cerebro connectivity. In Fig.2.c.ii, we show the group difference of oculo-cerebro connectivity. Here we list the HCPMMP1 labels of the regions included in clusters in the left hemisphere. The regions are ordered by their contribution to the cluster statistics. The cluster in the left hemisphere spans frequencies from 0.17 to 3.33 Hz ($F(2, 44)=3187.8$, $p<0.001$) and peaks in the left secondary auditory cortex (L-PBelt-ROI-lh). Download Figure 2-2, TIF file.

### Ocular speech tracking is not significantly modulated by intelligibility

To further investigate the functional properties of ocular speech tracking, we tested whether oculomotor activity is modulated by speech intelligibility. We propose that if there is a condition effect such that the acoustics of intelligible speech are tracked more strongly than those of unintelligible speech (i.e., 1-channel vocoded), then closed-eye ocular activity would be responsive to the intelligibility of speech. Conversely, if not, we suggest that closed-eye ocular activity tracks speech envelope modulations irrespective of intelligibility. In line with the latter, no significant clusters were found in the coherence effects for oculo-acoustic coherence (Fig. 1*a*.iii). To test for the absence of effects at 1 Hz (where the tracking peaked in Fig. 1*a*.iii), we performed a TOST. Pairwise comparisons between the three stimulus conditions did not reach the threshold for statistical equivalence at the 0.01 bound (natural vs. voc1: *p*_TOST_ = 0.055; natural vs. voc8: *p*_TOST_ = 0.180; and voc1 vs. voc8: *p*_TOST_ = 0.348). To rule out the possibility that ocular effects reflect volume conduction from auditory sources, and to validate our approach against prior evidence showing that neural speech tracking is modulated by intelligibility (Van Ackeren et al., 2018; Hauswald et al., 2022; Chen et al., 2023; Schmidt et al., 2023), we compared neural tracking across the three intelligibility conditions. Both coherence and mTRF analyses replicated the patterns of previously reported effects. By selecting the sensors that exhibit the strongest speech tracking (to show that auditory sources are not what drives ocular effects), we observed a neural condition effect between 0.33 and 1.17 Hz (*F*(2, 44) = 46.99, *p* < 0.05). Post hoc paired *t*-tests with Bonferroni correction revealed that the mean coherence between 0.33 and 1.17 Hz was significantly stronger for natural speech compared to 1-channel vocoded speech (*t*(46) = 5.65, *p* < 0.001, *Hedges*′ *g* = 0.86), and significantly stronger for 8-channel vocoded speech compared to 1-channel vocoded speech (*t*(46) = −6.38, *p* < 0.001, *Hedges*′ *g* = −0.81). No significant difference was found between the natural speech and the 8-channel vocoded speech conditions (*t*(46) = 1.89, *p* = 0.193, *Hedges*′ *g* = 0.23). This is also in line with the behavioral results by Van Ackeren et al. (2018), showing no behavioral difference between the natural and the 8-channel vocoded condition. A similar pattern was observed for the mTRF approach (extended data; Fig. 1-1). This graded pattern replicates previous evidence (Chen et al., 2023; Schmidt et al., 2023) showing that cortical entrainment to speech acoustics is enhanced by intelligibility. This pattern is not present in the ocular data, suggesting that ocular tracking is reflective of a different mechanism of the perceptual system than neural tracking.

### Ocular tracking of acoustics changes as a function of visual experience

As the mechanisms underlying oculomotor tracking of speech acoustics remain unclear, we compared eye movements in early blind, late blind, and sighted participants to assess whether this phenomenon depends on visual experience or instead reflects hard-wired, experience-independent mechanisms. Coherence analysis revealed a significant main effect of group in the 5.5–6.33 Hz frequency range (*F*(2, 44) = 23.95, *p* = 0.014; Fig. 1*a*.ii), with sighted participants showing stronger ocular speech tracking than both blind groups. Post hoc group comparisons at 6 Hz showed significant differences between the early blind and the sighted control groups (*t*(85.24) = −2.54, *p*_Bonf_ = 0.039, *Hedges*′ *g* = −0.53) and between the sighted control group and the late blind group (*t*(78.87) = 2.91, *p*_Bonf_ = 0.014, *Hedges*′ *g* = 0.62). However, no significant difference was found between the early blind and the late blind groups (*t*(96.30) = 0.28, *p*_Bonf_ = 1.000). Comparing the ocular coherence at 6 Hz of the presented speech envelope to the control envelope, there is increased tracking for the control group in the presented acoustics (*t*(13) = −2.49, *p* = 0.027, *Hedges*′ *g* = −0.79, *BF*_10_ = 2.54). In contrast, no significant differences were found for the Early Blind group (*t*(16) = 0.14, *p* = 0.891, *Hedges*′ *g* = 0.04, *BF*_10_ = 0.25) or the Late Blind group (*t*(15) = 0.22, *p* = 0.832, *Hedges*′ *g* = 0.08, *BF*_10_ = 0.26). To further validate these null results, we performed Equivalence Testing (TOST) with a bound of 0.1. The tests confirmed that for both the Early Blind group (*p*_TOST_ < 0.001) and the Late Blind group (*p*_TOST_ < 0.001), the tracking values for the actual (presented) and a non-presented control envelope were statistically equivalent. Importantly, at ∼1 Hz, the frequency at which we observed a tracking effect in the previous step (Fig. 1*a*.i), no group differences were found. This was again tested using TOST for coherence at 1 Hz, and all pairwise comparisons between the three groups (early blind, late blind, and sighted controls) met the criterion for equivalence. This analysis provides strong statistical evidence for the absence of a difference in the overall 1 Hz ocular coherence between all three groups. Early blind versus sighted controls ( ${\text{CI}}_{90\%}=[-0.0113,0.0063]$); early blind versus late blind ( ${\text{CI}}_{90\%}=[-0.0083,0.0056]$); and sighted controls versus late blind ( ${\text{CI}}_{90\%}=[-0.0078,0.0101]$). This pattern suggests the involvement of two distinct underlying mechanisms: one at ∼1 Hz, present regardless of visual experience or sightedness, and another one at ∼6 Hz, observed only in sighted individuals. For the mTRF reconstruction accuracies, no significant main effect of group (*F*(44, 2) = 0.27, *p* = 0.765, $\eta_{g}^{2}=0.006$) was observed (Fig. 1-1. i.c). To ensure that the neural and ocular effects are distinct, the same statistics were conducted for auditory sensors, where no significant clusters appeared and the pattern differs from the ocular one (Fig. 1*b*.ii). In none of the cluster permutation ANOVAs (ocular coherence, neural coherence, neural mTRF reconstruction accuracy) the interaction (group * condition) revealed significant clusters. Also, for the mTRF of the eye data, no statistically significant interaction between group and condition ( $F(4,88)=1.36,p=0.256,\eta_{p}^{2}=0.040$) was found.

### Strongest oculo-cerebral connectivity patterns in late blind individuals

At ∼1 Hz, ocular tracking was strongest compared to the control envelope, and at ∼6 Hz, the group difference was strongest. Therefore, we investigated the connectivity between ocular and neural activity at both frequencies of interest (1 and 6 Hz). Using imaginary coherence, across the two frequencies of interest, we observed distinct patterns of oculo-cerebral connectivity: at 1 Hz, left temporal and inferior frontal regions appeared to follow the eyes, whereas at 6 Hz, the left temporal and inferior frontal regions preceded the eyes. Note that directionality should be interpreted with caution (see Methods, Coherence section). To compare the three groups in this measure, we used the absolute value of the imaginary coherence, focusing on the strength rather than the directionality of the connectivity, and ran an ANOVA. *F*-values across all parcellated areas for the frequencies, where we found the ocular effects, are illustrated in Figure 2*b*. Cluster-based permutation tests comparing oculo-cerebral connectivity revealed two widespread clusters. In the left hemisphere, the effect spans frequencies from 0.17 to 3.33 Hz (*F*(44, 2) = 3187.8, *p* < 0.001), including multiple regions (Figs. 2*c*.i, 2-2) and peaking in the left secondary auditory cortex (L-PBelt-ROI-lh). In the right hemisphere, the cluster peaks in occipital cortex (V7: R-V7-ROI-rh), in slightly higher frequencies, from 2 to 3.6 Hz (*F*(44, 2) = 608.07, *p* < 0.01); all regions are shown in Figure 2*c*(ii) and listed in Figure 2-1. For these regions, we plotted the full frequency range (0.16–8 Hz) in Figure 2*c*.i,ii (left panel) and marked the previously on whole-brain level encountered cluster in gray. Finally, to test whether the enhanced oculo-cerebral connectivity or the neural tracking activity is driving the ocular tracking of the envelope, we compared neural envelope coherence across groups within the data-driven regions. No significant group differences were observed (Fig. 1*a,b*, right panel). This shows that the stronger oculo-cerebral connectivity observed in the late blind group is not leading to stronger neural speech tracking.

## Discussion

Here, we present the first study on whether visual input is needed for the oculomotor system to track incoming auditory information, to characterize how oculomotor activity changes as a function of speech intelligibility, and to investigate oculo-cerebral connectivity to explore which neural systems drive ocular tracking. We compared ocular and neural tracking in early blind, late blind, and sighted individuals while they listened to continuous, narrative speech. Our results show that closed eyes track the speech envelope, reflected in a low-frequency (∼1 Hz) effect across all groups and a high-frequency (∼6 Hz) effect only in sighted individuals. We suggest this pattern to reflect two different processes of ocular speech tracking—a hard-wired low-frequency process that is independent of visual experience and a high-frequency one only employed in a functionally intact visual system. In contrast to neural tracking, which has previously been shown to modulate with speech intelligibility (Hauswald et al., 2022; Chen et al., 2023; Schmidt et al., 2023), ocular tracking does not. Thus, we propose ocular speech tracking to reflect a functionally different mechanism of speech processing than neural speech tracking.

### Hard-wired low-frequency ocular tracking

We show that low-frequency (∼1 Hz) oculomotor activity in early blind, late blind, and sighted individuals tracks the speech envelope, even in the absence of visual input, with closed eyes (Fig. 1*a*.i; 1 Hz peak). This shows that ocular speech tracking is not dependent on changing retinal input, which would be the result of moving the eyes when they are open. The finding that ocular speech tracking is present across all groups additionally suggests that it does not occur as a consequence of previous exposure to audiovisual speech, for example, by interacting with a speaker. Whether ocular speech tracking is hard-wired or develops in response to audiovisual speech exposure was answered in part by one of our previous studies (Benz et al., 2025). There, we demonstrated enhanced ocular tracking in late-deaf compared to hearing individuals. Stronger visual involvement in late-deaf individuals, who rely more on lip movements as a compensatory strategy for reduced hearing abilities, may therefore contribute to increased ocular tracking. Its absence in early-deaf individuals facing visual speech (lip movements) suggests that it needs auditory exposure to speech to develop. Complementing this, the present results suggest that while auditory input is necessary for the emergence of ocular tracking, visual input is not—challenging the idea that ocular speech tracking around 1 Hz depends on audiovisual learning and instead supporting the notion of a hard-wired mechanism likely involved in low-frequency temporal parsing of continuous speech.

This slow tracking of speech peaking at 1 Hz is mainly involved in segmenting speech without periodic activity based on speech onsets, such as the beginning of a sentence (Chalas et al., 2023). It could also reflect language-independent intonation units, which are reflected in the envelope and generate a stable low-frequency rhythm at 1 Hz (Inbar et al., 2020, 2025). Investigating oculo-cerebral activity, instead of a strong occipital involvement, we show left temporal oculo-cerebral connectivity at ∼1 Hz (Fig. 2*a*).

Even though we caution against strong interpretation of directionality, we would like to note that, surprisingly, the auditory signal perceived via the ears is phase-preceding in the eyes relative to left temporal regions at low frequencies. While functionality of ocular speech tracking is still unclear, one possibility could be that direct eye-to-ear connections (Lovich et al., 2023)—which are also present without any visual input (Abbasi et al., 2025)—account for this low-frequency effect. In this way, eye movements may help to structure auditory processing at language-independent intonation units present at 1 Hz (Inbar et al., 2025) and reflect an internal model of speech, potentially explaining why auditory (Benz et al., 2025), but not visual, experience is required. Moreover, we find that activity in medial frontal and parietal regions, which are suggested to drive attentional processes, precedes ocular activity at 1 Hz. This is in line with findings suggesting that attention drives ocular speech tracking (Gehmacher et al., 2024a; Schubert et al., 2024). This ocular speech tracking effect at 1 Hz, independent of visual status and intelligibility, may reflect a combination of top-down attentional processes and bottom-up processing, capturing both low-level acoustic features (Chalas et al., 2023) and intonation units (Inbar et al., 2025).

### High-frequency ocular tracking develops in response to previous exposure to audiovisual speech

As discussed above, a central question of the present study was whether ocular speech tracking is a hard-wired phenomenon or shaped by visual experience. Our results suggest that both factors play a role. Although the three groups do not differ in the low-frequency (∼1 Hz) effect, sighted controls show increased tracking in the high-frequency (∼6 Hz) effect compared to the blind groups. The speech feature supported at ∼6 Hz is the syllable rate, which might be reflected in this effect. The enhanced tracking in the sighted group suggests that participants with intact eyesight show stronger ocular speech tracking, even when blindfolded. Syllabic transition rates expressed in lip movements and speech might be learned by audiovisual experience. Because sighted individuals typically integrate audiovisual information during speech perception, auditory input may also be conveyed to the oculomotor system. In the present data the mean syllable rate is approximately 6 Hz, suggesting that auditory processing in temporal regions may support local computations and, in turn, drive or support ocular tracking of speech. Consequently, greater involvement of the visual system predicts stronger phase locking of eye movements to the speech envelope. Coherent with previous research suggesting bidirectional oculo-cerebral connectivity (Madsen and Parra, 2024), we find that a left temporal network precedes ocular activity at ∼6 Hz, indicating that these processes may enhance ocular tracking at this frequency in sighted individuals. This (∼6 Hz) peak at the syllable rate in sighted individuals may reflect functional involvement of the oculomotor system in auditory processing. Importantly, this effect is unlikely to be explained by audiovisual experience alone, as late blind participants would be expected to show similarly increased tracking at 6 Hz if this were the case; instead, it appears to depend on visual experience. Although coherence reveals increased tracking at 6 Hz, the mTRF approach did not yield a statistically significant effect. This likely reflects methodological differences, as mTRF relies on a single summary metric (prediction accuracy), whereas coherence provides frequency-resolved information. Here, the coherence effect is driven by a narrow band (e.g., 6 Hz) that is not sufficiently powerful to significantly modulate the single-value reconstruction accuracy.

### Oculomotor activity of blindfolded individuals tracks speech acoustics independently of intelligibility

In line with previous research suggesting that ocular speech tracking reflects an attentional mechanism supporting challenging listening situations, posing the idea that listening to vocoded speech might enhance ocular speech tracking (Gehmacher et al., 2024a; Schubert et al., 2024; Benz et al., 2025), we expected to find ocular tracking to change as a function of speech intelligibility. Unlike neural tracking, ocular tracking was not significantly affected by the stimulus condition. That said, we were unable to prove the absence of an effect, as the data did not meet the criteria for statistical equivalence. Our results show the expected differences of higher neural tracking with higher intelligibility, confirming previous findings (Chen et al., 2023; Schmidt et al., 2023). In general, acoustically intact speech elicits stronger neural tracking and leads to shifts in temporal and spectral signal characteristics (Chen et al., 2023; Schmidt et al., 2023). It is possible that *we do not find a vocoding effect in* ocular speech tracking as a consequence of a lower signal to noise ratio in the eye data. However, previous work has reported a negative relationship between comprehension and ocular speech tracking (Schubert et al., 2024)—in other words, lower ocular tracking when comprehension is higher—which leads us to speculate that the eyes may not show stronger tracking with better comprehension, as is commonly reported in neural data. This could be the reason for why we do not observe the typical vocoding effect in the ocular data. Additionally, the modulation of ocular tracking in response to speech intelligibility has so far only been investigated using a multi-speaker experimental design (Gehmacher et al., 2024a), which elicited higher ocular speech tracking of the attended speech stream compared to a single-speaker setting. While the precise relationship between ocular and neural tracking of acoustic signals is not yet fully understood, evidence suggests bidirectional connections between the eyes and the brain (Madsen and Parra, 2024), which would provide a mechanistic basis for coordinated speech tracking. Applying the logic of these findings to our present results, we propose that neural circuits in relevant processing areas track the higher linguistic structures of speech, while the eyes track attended low-level acoustic features or intonation units (Inbar et al., 2020, 2025) and might track sounds in general, thus responding more to attentional focus rather than intelligibility per se. This enables the integration of the different functional mechanisms of neural and ocular tracking into one coherent framework of speech tracking.

### Increased functional connectivity between eyes and brain sources in late blind individuals does not lead to enhanced envelope tracking

Sighted participants showed the highest ocular speech tracking values at ∼6 Hz, which intuitively led to the expectation of enhanced connectivity with left temporal regions at this frequency. Contrary to this assumption, however, late blind individuals show increased oculo-cerebral coherence compared to both other groups. This increased coupling may reflect enhanced connectivity between auditory and visual systems following late-onset sensory loss. Stronger auditory-to-visual connectivity has previously been reported in late blind individuals (Sabbah et al., 2016), and stronger inter-modal connectivity has been shown in late-deaf individuals with (Fullerton et al., 2022) and without (Shang et al., 2020) cochlear implants. Thus, vision loss at a later stage of development might strengthen oculo-cerebral connectivity. Importantly, this increase does not translate into stronger ocular envelope tracking (Fig. 1*a*.ii) or enhanced neural tracking (Fig. 1*b*.ii). While enhanced auditory tracking in occipital regions could, in principle, be driven by eye movements, we did not observe increased occipital speech tracking in the blind groups. Moreover, oculo-cerebral connectivity did not modulate envelope tracking strength. Together, these results suggest that connectivity and tracking reflect independent processes, supporting a distinct interpretation of the connectivity effect. In line with Koba et al. (2024), our findings further indicate that oculo-cerebral circuitry is retained in early blind individuals.

### Source-reconstructed ocular activity

Because gold-standard eye-tracking methods were impossible due to the mandatory blindfolding and the lack of established validation data, we utilized a robust, anatomically constrained alternative. We did successfully identify an ICA eye component for every subject, either based on the topographies (template matching, component topography symmetry) or the time-series (correlation with frontal channels). This observation aligns with previous studies showing that blinks and saccades persist in individuals who are blind or blindfolded (Händel et al., 2025). Furthermore, we show distinct patterns of the ocular and neural data: both the group and the condition effect in the neural tracking differ from those in the eyes, suggesting that they reflect distinct underlying processes. To ensure this, in addition to the auditory coherence effects (Fig. 1ii), we plotted the patterns of the sensors with the strongest effects in the extended data. Moreover, the shape of our mTRF weights is consistent with previous findings from eye-tracking data analysis (Schubert et al., 2024), but not with those present in the neural data (extended data).

### Limitations

Importantly, current eye-tracking methods are optimized for sighted individuals, limiting the precision of gaze-based measures in blind populations. Therefore, we extracted ocular activity using source reconstruction, which is not a well-established eye-tracking method. So, we cannot disentangle the direction (horizontal vs. vertical) of the eye movements, nor reliably identify saccades or blinks, as eye movement patterns in the blind groups showed a high variety. Consequently, we refer to all recorded ocular activity collectively as eye movements. To disentangle this, future research should aim to develop improved tracking techniques tailored to blind individuals, extracting saccades and blinks (Händel et al., 2025) or infrared eye-tracking methods also working with closed eyes (Ben Barak-Dror et al., 2024). Gehmacher et al. (2024a) and Schubert et al. (2024) suggest a functional or compensatory role for ocular tracking, while Madsen and Parra (2024) propose bidirectional brain-body interactions. Although ocular tracking could be viewed as epiphenomenal, the divergent patterns observed here between neural and ocular measures indicate that it is not merely a byproduct of neural activity. This dissociation suggests that ocular tracking may serve a distinct role, though future causal testing is required to confirm its specific contribution to perception.

### Conclusion

Our findings provide the first evidence that the oculomotor system tracks auditory information without visual input. Low-frequency (∼1 Hz) ocular activity aligned with the speech envelope across all groups indicates a hard-wired mechanism supporting auditory processing. In contrast, high-frequency (∼6 Hz) tracking emerged only in sighted individuals, suggesting an experience-dependent process shaped by audiovisual integration. Unlike neural speech tracking, ocular data did not reveal an effect nor the absence of an effect of speech intelligibility. Enhanced oculo-cerebral coupling in late blind individuals might support enhanced multisensory integration following sensory loss, but does not lead to increased ocular tracking. Together, these results expand current models of active sensing by demonstrating that eye movements, independent of vision, contribute to the temporal organization of auditory processing, positioning the eyes as active agents in the perception of speech.

## Code Availability

GitLab: gitlab.com/KajaRosa/kb_blindaudio.

## Data Availability

The authors acknowledge the Austrian NeuroCloud (https://anc.plus.ac.at/), hosted by the University of Salzburg and funded by the Federal Ministry of Education, Science and Research (BMBWF), for providing a FAIR-compliant research data repository. Find the data on request on the ANC: bids-datasets.data-pages.anc.plus.ac.at/auditory/ma_blindaudio/.

## Synthesis

Reviewing Editor: Niko Busch, Westfalische Wilhelms-Universitat Munster Decisions are customarily a result of the Reviewing Editor and the peer reviewers coming together and discussing their recommendations until a consensus is reached. When revisions are invited, a fact-based synthesis statement explaining their decision and outlining what is needed to prepare a revision will be listed below. The following reviewer(s) agreed to reveal their identity: Flor Kusnir. Note: If this manuscript was transferred from JNeurosci and a decision was made to accept the manuscript without peer review, a synthesis may not be available.

# Synthesis

Your manuscript has now been evaluated by two experts. Both reviewers find the study interesting and methodologically innovative, and they agree that it makes a valuable contribution to the literature on speech tracking and brain-body interactions. Reviewer 1 notes that the concerns raised in the previous round have been addressed and recommends acceptance.

Reviewer 2 is also very positive about the work and provides several constructive suggestions aimed primarily at clarifying aspects of the methodology and interpretation, particularly regarding the validation of the eye-movement reconstruction approach and the presentation of some analyses. These points should be relatively straightforward to address and are intended to further strengthen the manuscript. Since these comments parallel Reviewer 1's earlier concerns about ocular tracking in the absence of EOG or eye tracking, I believe they are worth careful consideration and should be addressed in the revision.

Please revise the manuscript accordingly and provide a point-by-point response to the reviewer's comments.

# Reviewer 1

This research is a continuation of recent work examining brain-body interactions during narrative speech. The main finding is that one aspect of ocular information (1 hz oculomotor activity) tracks the speech envelope in sighted, early, and late-blind individuals, but that the groups differed at 6 hz, putatively linked to syllable rate. It demonstrates that the oculomotor system is capable of tracking auditory speech information even in the absence of retinal processing.

The authors have addressed my previous comments. While I still believe that validation against a gold-standard measure of eye movements (such as concurrent eye-tracking or EOG) would meaningfully strengthen confidence in these results, I recognize that requiring additional data collection at this stage would be disproportionate to the scope of a revision. The authors have taken reasonable steps to address the concern analytically, and the discussion now gives readers sufficient information to evaluate the robustness of the findings for themselves. I have no further major concerns and recommend the manuscript for acceptance.

# Reviewer 2

From a methodological perspective, the manuscript uses sophisticated and innovative methods to reconstruct eye movements. Though they are not fully validated in this study, I do believe that the authors do an adequate job in reporting and justifying their approach. The methods seem sound and would be of potential interest to a wide readership. From a theoretical perspective, their findings with respect to ocular tracking of the speech signal are of particular interest on several levels. First, they contribute to our understanding of the underlying mechanisms by investigating the contribution of visual experience to speech-related eye movements. Second, from a linguistic perspective, their findings are meaningful and in light of recent studies suggesting the importance of both 1-hz and 6-hz tracking of the speech envelope, provide support for the eyes as participating in meaningful temporal parsing of speech and fits with active sensing frameworks and motor-perceptual loops.

The overarching aim of this very interesting study was to assess 1) whether visual input and experience are needed for ocular speech tracking, and 2) whether ocular tracking varies with speech intelligibility. In addition, (3) the authors performed eye-brain connectivity analyses to reveal the brain areas involved and to infer directionality.

Since participants were blindfolded and had their eyes closed, the authors could not use traditional eye tracking and so took an innovative approach by source reconstructing the eye movements. I find this approach intriguing, valid and valuable for setups in which gold-standard eye tracking methods are not possible. I thought the study was theoretically interesting - particularly the 1 Hz findings, which have recently shown to be especially meaningful in speech tracking and show features of universality in line with the authors' findings.

Before commenting on the rest of the manuscript, I have a couple of questions regarding this approach.

Methodological Questions:

Q1) I saw that a previous reviewer already asked whether the authors performed ICA and I see that the authors replied with a figure of the ICA components for each participant. Looking through them, many of them do not look like typical eye movement ICA components, and many of them also seem to be very late components (judging from the number on each ICA component label) meaning that they account for very, very little variance in the signal, also very atypical of eye movements which are typically huge compared to neural data. While I understand that the combination of blind participants, a blindfold, and closed eyes probably results in smaller and less controlled eye movements and messier ICA components, I would anyway expect the presence of eye movements to result in relatively clear ICA components, at least for a good handful of participants. In fact, it would have been helpful to validate these ICA components with their respective timecourses (at least for a select few from each experimental group). Eye movements have very clear signatures so rather than correlating topographies across participants (as you currently do), simply showing some ICA components along with their timecourse would be validating.

I am not suggesting that the authors add this to the main manuscript, but it would be validating and reassuring to have this ICA figure along with the timecourses in the supplemental information. I am interested in the authors' thoughts regarding this suggestion and - if you think otherwise - I am open to hearing why.

Q2) The authors extract the first PCA component to reconstruct eye movements, which I would expect would likely reflect eye movements. I believe this to be a sound method, but since it is quite innovative and rarely used (I don't know any other papers doing this), I was wondering whether you performed other sanity checks before continuing with your analyses. For example, did you look at the topographies associated with these PCA components (projected back to sensor space), just to see whether they look like typical ocular and not cortical topographies? Or perhaps correlating the reconstructed eye components with MEG sensors to see whether they peak in frontal channels (again, as a sanity check that the components truly reflect eye movements)? Overall, my question here is whether you performed any kind of sanity checks to further validate that the extracted signals likely reflect eye movements.

In support of your approach, I would like to highlight that your 6 Hz findings are quite encouraging. I would even argue that they almost act as a sort of internal control of your eye-reconstruction approach. The fact that they are present only in sighted individuals, that they are in line with established literature focusing on natural speech tracking (i.e., syllabic speech rhythms), and that they don't covary with intelligibility - together, these points suggest that the tested signal is 1) meaningful and 2) distinct from a brain signal (which would be expected to covary with intelligibility). Perhaps stating this clearly and explicitly would help alleviate methodological doubts that many readers will have, given the novelty and rarity of this approach, and given that it could not be validated with eye-tracking or ICA (more widely accepted).

General Questions:

Point 1) A clarification question: sometimes it is not clear whether statistics were run on the individual subject values, or on averages across groups? Were speech tracking and coherence performed over all subjects of one group, or per subject and then averaged? I assume most analyses here include the single-subject data but please clarify this point.

Point 2) The overall coherence in the ocular response to speech is compared to a control envelope (as seen in Fig.1ai), but since the authors are mainly interested in the contribution of visual experience to speech tracking, it is not clear to me why the analysis collapses the groups to begin with. The coherence by group analysis (as seen in Fig.1aii) does not test against a control envelope (across all frequencies, by group). I see that the authors responded to a previous concern by Reviewer 1 (Point 1) and tested against a control envelope at 6 Hz only (post-hoc), but this leaves me wondering why the entire range of frequencies was not tested against a control envelope (by group). Might the sighted group also show coherence (compared to a control envelope) in lower frequencies as well (not clear from the x-axis where the middle peak lies, but probably around 3 Hz)? Could the authors comment on this analytic decision? If you could clarify the logic here, then I don't necessarily believe that the analyses need to be redone.

Minor:

Could you specify somewhere in the manuscript (and/or figures) which of the two non-natural speech stimuli is most scrambled and difficult to understand (1 channel or 8 channel vocoded)?

Figure 1 would benefit from finer x-axis labels. I would suggest adding at least all odd numbers, or all frequencies from 1-6.

In Figure S1, it would be helpful to have a more complete caption. Some panel descriptions are currently missing (for example, for panel i, there is no description of subfigures a and b, only of subfigure c. It would be helpful to have a more complete caption in order to better guide the reader.

Line 407 of the Discussion: What do the authors mean by "...instead supporting the notion of a hard-wired mechanism involved in auditory processing"? The wording is vague. Please be more specific rather than merely writing "auditory processing."

A comment: the 1-Hz ocular tracking across groups is striking and very interesting. The authors cite Inbar, 2020, but there is an additional more recent study (Inbar 2025 in PNAS) that extends that work and suggests a universality of the 1-Hz intonation unit (across many languages worldwide). Those findings suggest that this low frequency (1 Hz) temporal structure might be foundational to language and cognition. The authors' findings (1-Hz tracking across visual experience conditions) really speak to this possibility, in my opinion, making your findings of potential interest to a wider (linguistic) readership.

Line 418 of the Discussion: "...suggesting an eye-ear connection that enables direct auditory information transmission (Lovich et al., 2023)." Could the authors elaborate in a sentence or two what they mean here? Are you suggesting that eye movements at 1 Hz might help structure auditory processing of speech?

As a more general comment, I would urge the authors to discuss inferred directionality more cautiously, since (as far as I know) imaginary coherence is less stable at low frequencies, and the fact that the brain/eye/speech envelope are all likely correlated, plus the shared stimulus drive - make directionality hard to infer. I do not think these analyses or interpretations need to be heavily edited - but a word of vigilance is needed here.

Line 430 - "participants with sighted eyes" - is this terminology commonly used? If not, I would replace with "participants with intact eyesight" or something like that because "sighted eyes" sounds a bit strange and unclear.

Lines 462-465 of the Discussion - "It is possible that no clear evidence... which would not speak for the same effect in the eyes as neurally observed." There are some phrasing issues here which make these sentences very difficult to follow or understand. When you write, "no clear evidence of ocular speech tracking" which analysis are you referring to? Intelligibility (as in the previous sentences) or do you mean something more general? Also, what does "a negative relationship" mean - could you clarify or spell it out? The last part of the phrase is also not clear - "...which would not speak for the same effect in the eyes as neurally observed." I would appreciate it if these sentences could be reworded for clarity.

Lines 474-475 Earlier you suggested that the 1 Hz tracking might be of Intonation Units, but these are not low-level acoustic features of speech like pitch and duration. They rather make use of low-level acoustic features and are thought to reflect information chunking. The lack of a finding between ocular speech tracking and intelligibility here is consistent with the possibility that the eyes track intonation units, especially given that there is evidence in the literature that intonation units can be identified by listeners even in languages they do not understand.

Line 505 - "Furthermore, we show distinct patterns of the ocular and neural data..." When the authors refer to the group and condition effect in the "neural tracking measure" - what are they referring to? There are many analyses in this work so specificity would be appreciated by the readers.

As a very minor point, the paper is generally well written, but there are tense errors and some awkwardness in phrasing throughout. If your team has an English editor on board, I would encourage a run through, as it would make for a smoother read.

## Author Response

Response to Reviewer 1 This research is a continuation of recent work examining brain-body interactions during narrative speech. The main finding is that one aspect of ocular information (1 hz oculomotor activity) tracks the speech envelope in sighted, early, and late-blind individuals, but that the groups dif- fered at 6 hz, putatively linked to syllable rate. It demonstrates that the oculomotor system is capable of tracking auditory speech information even in the absence of retinal processing. The authors have addressed my previous comments. While I still believe that validation against a gold-standard measure of eye movements (such as concurrent eye-tracking or EOG) would mean- ingfully strengthen confidence in these results, I recognize that requiring additional data collection at this stage would be disproportionate to the scope of a revision. The authors have taken rea- sonable steps to address the concern analytically, and the discussion now gives readers su!cient information to evaluate the robustness of the findings for themselves. I have no further major concerns and recommend the manuscript for acceptance.

We thank the reviewer again for their helpful input in the first round and appreciate the positive feedback on the article.

Response to Reviewer 3 From a methodological perspective, the manuscript uses sophisticated and innovative methods to reconstruct eye movements. Though they are not fully validated in this study, I do believe that the authors do an adequate job in reporting and justifying their approach. The methods seem sound and would be of potential interest to a wide readership. From a theoretical perspective, their findings with respect to ocular tracking of the speech signal are of particular interest on several levels. First, they contribute to our understanding of the underlying mechanisms by investigating the contribution of visual experience to speech-related eye movements. Second, from a linguistic perspective, their findings are meaningful and in light of recent studies suggesting the importance of both 1-hz and 6-hz tracking of the speech envelope, provide support for the eyes as participating in meaningful temporal parsing of speech and fits with active sensing frameworks and motor-perceptual loops.

The overarching aim of this very interesting study was to assess 1) whether visual input and experience are needed for ocular speech tracking, and 2) whether ocular tracking varies with speech intelligibility. In addition, (3) the authors performed eye-brain connectivity analyses to reveal the brain areas involved and to infer directionality.

Since participants were blindfolded and had their eyes closed, the authors could not use traditional eye tracking and so took an innovative approach by source reconstructing the eye movements. I find this approach intriguing, valid and valuable for setups in which gold-standard eye tracking methods are not possible. I thought the study was theoretically interesting - particularly the 1 Hz findings, which have recently shown to be especially meaningful in speech tracking and show features of universality in line with the authors' findings.

Before commenting on the rest of the manuscript, I have a couple of questions regarding this approach.

Methodological Questions Point 1: I saw that a previous reviewer already asked whether the authors performed ICA and I see that the authors replied with a figure of the ICA components for each participant. Looking through them, many of them do not look like typical eye movement ICA components, and many of them also seem to be very late components (judging from the number on each ICA component label) meaning that they account for very, very little variance in the signal, also very atypical of eye movements which are typically huge compared to neural data. While I understand that the combination of blind participants, a blindfold, and closed eyes probably results in smaller and less controlled eye movements and messier ICA components, I would anyway expect the presence of eye movements to result in relatively clear ICA components, at least for a good handful of participants. In fact, it would have been helpful to validate these ICA components with their respective timecourses (at least for a select few from each experimental group). Eye movements have very clear signatures so rather than correlating topographies across participants (as you currently do), simply showing some ICA components along with their timecourse would be validating. I am not suggesting that the authors add this to the main manuscript, but it would be validating and reassuring to have this ICA figure along with the time-courses in the supplemental information. I am interested in the authors' thoughts regarding this suggestion and - if you think otherwise - I am open to hearing why.

Response: We thank the reviewer for this important thought. Since the participants had their eyes closed, were blindfolded, and some of them were blind, it is reasonable to question whether they produced substantial eye movements at all. This is also crucial for the source reconstruction: if no eye movements occurred, none would be captured by the source reconstruction either. The issue was not an absence of eye movement-related ICA components, but rather that eye-movement patterns varied considerably both within and between the three groups, and the resulting ICA components were less "typical" than those commonly observed in sighted participants with their eyes open. Importantly, the within group di"erences were higher in the blind groups than in the sighted group. This underscores the advantage of source reconstruction, as it does not rely on typical patterns but instead on eye location, which remains consistent across participants. Due to signal length and the number of participants, we cannot present the whole time series for all participants. Instead, we now include a 30 second data segment for five participants per group in the supplementary materials.

Importantly, we would like to emphasize that ICA served here as a supportive validation step rather than the primary basis of our analysis. Its variability across participants underscores the advantage of the source-reconstruction approach, which does not rely on signal patterns but instead leverages the anatomical consistency of the eye location across individuals. Accordingly, our main conclusions are based on the source-reconstructed signals, which we consider more robust in this specific experimental context.

Revised Text (Supplements 2) Exemplary 30s ICA-Eye components Group Late lind Early Blind Control Figure 1: Representative ocular-related ICA components and corresponding time courses across groups. Example independent components classified as ocular­ related are shown for five participants per group (early blind, late blind, sighted controls), together with 30-second segments of their time courses. Note that the between subject variability within the blind groups was higher than in then sighted group, which was one reason we selecd source reconstruction. See Method­ s/MEG Data Preprocessing section for information on ICA-eye channel selection.

Point 2: The authors extract the first PCA component to reconstruct eye movements, which I would expect would likely reflect eye movements. I believe this to be a sound method, but since it is quite innovative and rarely used (I don't know any other papers doing this), I was wondering whether you performed other sanity checks before continuing with your analyses. For example, did you look at the topographies associated with these PCA components (projected back to sensor space), just to see whether they look like typical ocular and not cortical topographies? Or perhaps correlating the reconstructed eye components with MEG sensors to see whether they peak in frontal channels (again, as a sanity check that the components truly reflect eye movements)? Overall, my question here is whether you performed any kind of sanity checks to further validate that the extracted signals likely reflect eye movements.

Response: H¨andel et al. (2025) suggest that eye-movement patterns are similar in blind and sighted individuals. Our participants have diverse etiologies for their blindness; consequently, we cannot be certain of the specific eye-movement profiles to expect from each individual. To avoid the circular reasoning of looking for patterns we cannot definitively predict, we wanted to avoid to look at everyone's topography just to look for typical ocular patterns previously. However he reviewer raises a valid point, and we have now included topographies for all participants for both the coherence and mTRF results (Supplementary Figure iv.a and iv.b.). Both measures show the highest values at left and right frontal channels, consistent with ocular activity - providing a useful sanity check. We thank the reviewer for this suggestion.

Revised Text (Supplements 1 vi.a+b.) Figure 2: Added figures iv.a + b Point 3: In support of your approach, I would like to highlight that your 6 Hz findings are quite encouraging. I would even argue that they almost act as a sort of internal control of your eye-reconstruction approach. The fact that they are present only in sighted individuals, that they are in line with established literature focusing on natural speech tracking (i.e., syllabic speech rhythms), and that they don't covary with intelligibility - together, these points suggest that the tested signal is 1) meaningful and 2) distinct from a brain signal (which would be expected to covary with intelligibility). Perhaps stating this clearly and explicitly would help alleviate methodological doubts that many readers will have, given the novelty and rarity of this approach, and given that it could not be validated with eye-tracking or ICA (more widely accepted).

Response: We thank the reviewer for this encouraging comment. We think it's a great idea to include the syllable rate in this reasoning, which we did not do previously. It is true that in German, with a syllable rate of around 4 Hz, there are vocoding e"ects at the frequency of the syllables in neural data (Chen et al., 2023; Schmidt et al., 2023). However, for our data with Italian audiobooks and a syllable rate of 6 Hz, we do not see this on the neural level (see Figure S1.ii.a), even when we test only for 6 Hz; correcting for neighbouring sensors, we do not find an condition e"ect. Testing for the absence of an e"ect in the neural data (pre-selecting the auditory channels from Fig. 1.b.1): pairwise comparisons between the three stimulus conditions did not reach the threshold for statistical equivalence at the 0.01 bound (natural vs. voc8: pTOST = .238; natural vs. voc1: pTOST = .090; and voc1 vs. voc8: pTOST = .242). In other words, we find neither an e"ect nor the absence of an e"ect. In the ocular signal, pairwise comparisons between the three stimulus conditions did reach the threshold for statistical equivalence at the 0.01 bound (natural vs. voc8: pTOST = .005; natural vs. voc1: pTOST = .004; and voc1 vs. voc8: pTOST = .004). However, in the absence of evidence for a neural e"ect, we cannot take this data to validate the ocular source-reconstruction. We appreciate this suggestion it would have been a nice point to make, but the data do not support it here.

General Questions Point 1: A clarification question: sometimes it is not clear whether statistics were run on the individual subject values, or on averages across groups? Were speech tracking and coherence performed over all subjects of one group, or per subject and then averaged? I assume most analyses here include the single-subject data but please clarify this point.

Response: All analyses were performed at the single-subject level and averaged for plotting. We have now clarified this explicitly in the description of the statistical analyses.

Revised Text (Line 266-268) "All statistical analyses were performed on subject-level data, with each partici- pant contributing one observation per condition to the respective tests." Point 2: The overall coherence in the ocular response to speech is compared to a control envelope (as seen in Fig.1ai), but since the authors are mainly interested in the contribution of visual experience to speech tracking, it is not clear to me why the analysis collapses the groups to begin with. The coherence by group analysis (as seen in Fig.1aii) does not test against a control envelope (across all frequencies, by group). I see that the authors responded to a previous concern by Reviewer 1 (Point 1) and tested against a control envelope at 6 Hz only (post-hoc), but this leaves me wondering why the entire range of frequencies was not tested against a control envelope (by group). Might the sighted group also show coherence (compared to a control envelope) in lower frequencies as well (not clear from the x-axis where the middle peak lies, but probably around 3 Hz)? Could the authors comment on this analytic decision? If you could clarify the logic here, then I don't necessarily believe that the analyses need to be redone.

Response: We thank the reviewer for this thoughtful point and the opportunity to clarify the analytical rationale. Our primary objective was to determine whether visual experience modulates ocular speech tracking. Accordingly, the central statistical comparisons focused on between-group di"erences in tracking strength, rather than on the presence or absence of tracking per se. The comparison against a control envelope was therefore implemented as a validation step to establish that the observed coherence reflects coupling to a meaningful acoustic signal, rather than a general property of the data. While we found the 1 Hz e"ect across control groups, which was not the case for the 6 Hz e"ect, we tested this subsequently. To maintain the clarity of our primary findings and avoid over-complicating the narrative with non-hypothesized results, we have not included this analysis. Importantly, the conclusions of the study are based on the observed di"erences between groups in speech tracking. The control-envelope analysis served to establish that the signal reflects meaningful stimulus coupling, whereas the group comparison determines how this coupling varies depending on visual status. We have clarified this in the manuscript.

Revised Text (Line 289-291) "The comparison against a control envelope was used as a validation step to estab- lish stimulus-specific coupling, whereas within- and between-group statistics were focusing on di!erences in tracking of the presented speech envelope." Minor Point 1: Could you specify somewhere in the manuscript (and/or figures) which of the two non-natural speech stimuli is most scrambled and di!cult to understand (1 channel or 8 chan- nel vocoded)? Response: We thank the reviewer for this helpful suggestion, as this is an important information to communicate. We added this to the manuscript.

Revised Text (Line 127-131) "This manipulation preserved the overall amplitude envelope while gradually degrading spec- tral detail. In perceptual terms, the 8-channel version produced substantial vocal distortion without impairing intelligibility, whereas the 1-channel version rendered speech entirely unin- telligible. Thus, the 1-channel vocoded condition represents the most degraded and least intelligible stimulus." Point 2: Figure 1 would benefit from finer x-axis labels. I would suggest adding at least all odd numbers, or all frequencies from 1-6.

Response: We thank the reviewer for this suggestion. We agree that finer axis labeling can enhance readability. At the same time, increasing the number of labels substantially reduces visual clarity given the limited space of the figure. We therefore retained the current labeling, while ensuring that the frequencies of primary interest (in particular →1 Hz and →6 Hz) are clearly identifiable.

Point 3: In Figure S1, it would be helpful to have a more complete caption. Some panel descrip- tions are currently missing (for example, for panel i, there is no description of subfigures a and b, only of subfigure c. It would be helpful to have a more complete caption in order to better guide the reader.

Response: We thank the reviewer for this helpful comment and agree that the figure caption required clarification. In addition to completing the missing descriptions, we have revised the wording and structure to improve clarity and overall flow.

Revised Text (Supplements 1) "Figure S1. Ocular and neural speech tracking mTRF analyses and source recon- struction sanity checks. i. Ocular tracking mTRF results. a) Reconstruction accuracy (mean {plus minus} SE) of the mTRF model for the speech envelope, compared against a control envelope. Significant tracking is observed for the true envelope (***p < 0.001). b) Average temporal response function (TRF weights) across par- ticipants. Shaded areas indicate {plus minus} SE. Note that the sign of the mTRF weights is not interpretable, as the polarity of the underlying signals is arbitrary. c) Re- construction accuracy across groups (sighted controls, early blind, late blind) and conditions. A mixed-design ANOVA with the dependent variable Ocular Tracking and factors Group (between-subjects) and Condition (within-subjects) revealed no significant main e!ect of group (F (2, 44) = 0.27, p = .765, ω2 = 0.006), no signifi- cant main e!ect of condition (F (44) = 0.73, p = .485, ω2 = 0.009), and no significant interaction between group and condition (F (4, 88) = 0.93, p = .448, ω2 = 0.022). ii. To show maximal di!erences between groups and conditions, and to make sure that the ocular e!ects are not based on neural speech tracking e!ects we plot the sen- sors with uncorrected p < 0.01 (blue) in a time or frequency resolution. Caution: Plots do not do not represent cluster-corrected statistical e!ect. a) Coherence between selected sensors and the speech envelope at 1 Hz. b) Condition di!er- ences in mTRF weights for the selected sensors. c) Group di!erences in coherence spectra. d) Group di!erences in mTRF weights. iii. Source reconstruction sanity checks. a) Source-level coherence with the speech envelope at 1 Hz. b) Source- level mTRF reconstruction accuracy. c) Coherence between reconstructed ocular activity and neural data at →1 Hz. These analyses confirm the spatial separation and consistency of ocular and neural signals." Point 4: Line 407 of the Discussion: What do the authors mean by "...instead supporting the notion of a hard-wired mechanism involved in auditory processing"? The wording is vague. Please be more specific rather than merely writing "auditory processing." Response: We thank the reviewer for this valuable input. In line with the rest of our argumenta- tion in the paper, we here explicitly referred to the low-frequency temporal parsing of continuous speech.

Revised Text (Line 412-414) "Complementing this, the present results suggest that while auditory input is necessary for the emergence of ocular tracking, visual input is not - challenging the idea that ocular speech tracking around 1 Hz depends on audiovisual learning and instead supporting the notion of a hard-wired mechanism likely involved in low-frequency temporal parsing of continuous speech" Point 5: A comment: the 1-Hz ocular tracking across groups is striking and very interesting. The authors cite Inbar, 2020, but there is an additional more recent study (Inbar 2025 in PNAS) that extends that work and suggests a universality of the 1-Hz intonation unit (across many languages worldwide). Those findings suggest that this low frequency (1 Hz) temporal structure might be foundational to language and cognition. The authors' findings (1-Hz tracking across visual expe- rience conditions) really speak to this possibility, in my opinion, making your findings of potential interest to a wider (linguistic) readership.

Response: We thank the reviewer for this insightful comment and for highlighting the recent work by Inbar (2025). We agree that the robust →1 Hz ocular tracking observed across groups is consistent with the idea of a universal low-frequency temporal structure in speech, potentially reflecting language-independent intonation units. We have now incorporated this perspective into the Discussion and expanded our interpretation of the 1 Hz e"ect accordingly (see revised Discus- sion (Line 421-437), next point). In particular, we relate our findings to the proposed universality of low-frequency structure in speech and discuss how ocular tracking at this frequency- indepen- dent of visual experience- may reflect a fundamental mechanism supporting speech segmentation and auditory processing. We agree that this connection broadens the relevance of our findings, and appreciate this input a lot.

Point 6: Line 418 of the Discussion: "...suggesting an eye-ear connection that enables direct auditory information transmission (Lovich et al., 2023)." Could the authors elaborate in a sentence or two what they mean here? Are you suggesting that eye movements at 1 Hz might help structure auditory processing of speech? Response: We thank the reviewer for raising this point. We are happy to clarify it further, as we consider it important and would like to emphasize it. We have rewritten the entire paragraph, also incorporating the points raised in the previous question.

Revised Text (Line 421-437) " This slow tracking of speech peaking at 1 Hz is primarily involved in segmenting speech without periodic activity based on speech onsets, such as the beginning of a sentence (Chalas et al., 2023). It could be also reflecting language independent intonation units, which are reflected in the envelope and generate a stable low frequency rhythm at 1 Hz (Inbar et al., 2020, 2025). Investigating oculo-cerebro activity, instead of a strong occipital involvement, we show left-temporal oculo-cerebral connectivity at →1 Hz (see Fig. 2.a). This suggests that slow stimulus-locked eye movements support speech processing in auditory regions. Further, ocular activity precedes activity in left-temporal regions. suggesting an eye-ear connection that enables direct auditory information transmission Even though we caution against strong interpretation of directionality, we would like to note that, surprisingly, the auditory signal perceived via the ears is phase-preceding in the eyes relative to left temporal regions at low frequencies. While functionality of ocular speech tracking is still unclear, one possibility could be that direct eye-to-ear connections (Lovich et al., 2023) - which are also present without any visual input (Abbasi et al., 2025)- account for this low-frequency e!ect. In this way, eye movements may help to structure auditory processing at language-independent intonation units present at 1 Hz (Inbar et al., 2025) and reflect an internal model of speech, potentially explaining why auditory, but not visual (Benz et al., 2025), experience is required. Moreover, we find that activity in medial frontal and parietal regions, which are suggested to drive attentional processes, precedes ocular activity at 1 Hz. This is in line with findings suggesting that attention drives ocular speech tracking (Gehmacher, Schubert, Schmidt, et al., 2024; Schubert et al., 2024). This ocular speech tracking e!ect at 1 Hz, independent of visual status and intelligibility, may reflect a combination of top-down attentional processes and bottom-up processing, capturing both low-level acoustic features (Chalas et al., 2023) and intonation units (Inbar et al., 2025). " Point 7: As a more general comment, I would urge the authors to discuss inferred directionality more cautiously, since (as far as I know) imaginary coherence is less stable at low frequencies, and the fact that the brain/eye/speech envelope are all likely correlated, plus the shared stimulus drive - make directionality hard to infer. I do not think these analyses or interpretations need to be heavily edited - but a word of vigilance is needed here.

Response: We appreciate the reviewer drawing our attention to this. They are totally right, and we are aware that imaginary coherence is not the standard method to investigate directionality. We added our concerns to the Coherence Methods part, and mentioned when discussing the results, that they should be interpreted with caution. "These were analyzed in a descriptive manner, and, we advise caution regarding the interpretation of directionality. Imaginary connectivity is a circular measure that we used to show both the negative and positive phase lag. At 6 Hz, the 166 ms cycle length is shorter than the 250 ms ocular response time reported by Gehmacher, Schubert, Kaltenmaier, et al., 2024. This introduces potential phase-wrapping issues, as a response occurring at 250 ms would exceed a full cycle, making it mathematically di"cult to distinguish a true lead from a lag. Nevertheless, as the e!ect remains robust across the group average and is highly compelling, we have chosen to report these findings here. " Revised Text (Line 365-366) Note that directionality should be interpreted with caution (see Methods Coher- ence section).

Revised Text (Line 421-423) Even though we caution against strong interpretation of directionality, we would like to note that, surprisingly, the auditory signal perceived via the ears is phase- preceding in the eyes relative to left temporal regions at low frequencies.

Point 8: Line 430 - "participants with sighted eyes" - is this terminology commonly used? If not, I would replace with "participants with intact eyesight" or something like that because "sighted eyes" sounds a bit strange and unclear.

Response: Done.

Revised Text (Line 446) "The enhanced tracking in the sighted group suggests that participants with sighted eyes intact eyesight show stronger ocular speech tracking, even when blindfolded. " Point 9: Lines 462-465 of the Discussion - "It is possible that no clear evidence... which would not speak for the same e"ect in the eyes as neurally observed." There are some phrasing issues here which make these sentences very di!cult to follow or understand. When you write, "no clear evidence of ocular speech tracking" which analysis are you referring to? Intelligibility (as in the previous sentences) or do you mean something more general? Also, what does "a negative relationship" mean - could you clarify or spell it out? The last part of the phrase is also not clear - "...which would not speak for the same e"ect in the eyes as neurally observed." I would appreciate it if these sentences could be reworded for clarity.

Response: We appreciate the reviewer's thoughtful comment. Our original wording may have been overly cautious and did not clearly distinguish between an absence of evidence for an e"ect and evidence for no e"ect. We have revised the text to clarify this distinction. "It is possible that we do not find a vocoding e!ect in ocular speech tracking as the consequence of a lower signal to noise ratio in the eye data. However, previous work has reported a negative relationship between comprehension and ocular speech tracking (Schubert et al., 2024) - in other words, lower ocular tracking when comprehension is higher - which leads us to speculate that the eyes may not show stronger tracking with better comprehension, as is commonly reported in neural data. This could be the reason why we do not observe the typical vocoding e!ect in the ocular data. " Point 10: Lines 474-475 Earlier you suggested that the 1 Hz tracking might be of Intonation Units, but these are not low-level acoustic features of speech like pitch and duration. They rather make use of low-level acoustic features and are thought to reflect information chunking. The lack of a finding between ocular speech tracking and intelligibility here is consistent with the possibility that the eyes track intonation units, especially given that there is evidence in the literature that intonation units can be identified by listeners even in languages they do not understand. Response: As the reviewer points out, both interpretations - speech segmentation based on non-periodic onsets and the tracking of language-independent intonation units - are plausible and, importantly, not mutually exclusive. With the current analysis, we cannot make a definitive statement in favor of one over the other. However, the fact that ocular tracking at 1 Hz is also present in the vocoded conditions, where intelligibility is reduced but low-level acoustic structure is preserved, is consistent with both accounts.

Revised Text (Line 492-495) "Applying the logic of these findings to our present results, we propose that neural circuits in relevant processing areas track the higher linguistic structures of speech, while the eyes track attended low-level acoustic features or intonation units (Inbar et al., 2020, 2025) and might track sounds in general, thus responding more to attentional focus rather than intelligibility per se." Revised Text (Line 434-437) "This ocular speech tracking e"ect at 1 Hz, independent of visual status and intelligibility, may reflect a combination of top-down attentional processes and bottom-up processing, capturing both low-level acoustic features (Chalas et al., 2023) and intonation units (Inbar et al., 2025)." Point 11: Line 505 - "Furthermore, we show distinct patterns of the ocular and neural data..." When the authors refer to the group and condition e"ect in the "neural tracking measure" - what are they referring to? There are many analyses in this work so specificity would be appreciated by the readers.

Response: We thank the reviewer for this constructive remark. It is true that we test the e"ects only once, using coherence for the auditory channels. This is the neural e"ect that can be interpreted. In the supplementary materials, we plot the sensors with the strongest e"ects; the purpose of these plots is to distinguish the neural e"ects from the ocular ones. They serve as sanity checks to demonstrate distinct patterns of neural and ocular e"ects, but we would not interpret them as standalone e"ects per se - we stated this clearer in the manuscript now.

Revised Text (Line 523-527) "Furthermore, we show distinct patterns of the ocular and neural data: both the group and the condition e"ect in the neural tracking di"er from those in the eyes, suggesting that they reflect distinct underlying processes. To ensure this, in addition to the auditory coherence e!ects (see Fig. 1ii), we plotted the patterns of the sensors with the strongest e!ects in the supplemental material." Point 12: As a very minor point, the paper is generally well written, but there are tense errors and some awkwardness in phrasing throughout. If your team has an English editor on board, I would encourage a run through, as it would make for a smoother read.

Response: We thank the reviewer for this helpful observation. We have carefully proofread the manuscript to correct tense inconsistencies and improve phrasing and some additional small changes like typos, capitalizations, commas, etc.

Revised Text (Proof reading over the whole manuscript) "Line 58: Although controlling for eye movements did not explain account for the observed neural e"ects in that work, in auditory speech, bidirectional interactions between oculomotor and neural systems (Gehmacher, Schubert, Schmidt, et al., 2024; Madsen &Parra, 2024) appear to play an important role in ocular speech tracking. Establishing how ocular activity couples with the brain during listening to speech could speech perception may clarify whether eye movements are driven by top-down cortical control or contribute provide bottom- up signals input to auditory processing in the absence of visual input.

Line 65: Generally, early-blind individuals are known to have superior hearing abilities compared In line with this, enhanced hearing abilities in this population in com- parison to sighted individuals have been reported , which is associated with activation in the visual cortex (Sabourin et al., 2022), which is also involved in the processing of spoken words and linked to activation in the visual cortex (Sabourin et al., 2022), which is likewise engaged in spoken word processing (Matuszewski et al., 2025).

Line 303: To rule out the possibility that ocular e"ects reflect volume conduction from audi- tory sources, and to validate our approach against prior evidence showing that neural speech tracking is modulated by intelligibility (Chen et al., 2023; Hauswald et al., 2022; Schmidt et al., 2023; Van Ackeren et al., 2018), we compared neural data tracking across the three intelligibility conditions. Both coherence and mTRF analyses confirmed the directionality of the replicated the patterns of previously reported results e!ects. By selecting the sen- sors that exhibit the strongest speech tracking (to show that auditory sources are not what drives ocular e!ects), we observed a neural condition e"ect between 0.33 and 1.17 Hz (F (44, 2) = 46.99, p< 0.05).

Line 370: The Cluster-based permutation tests comparing the oculo-cerebral connectivity revealed 2 two widespread clusters. On the left side In the left hemisphere, it starts at the e!ect spans frequencies from 0.167 to and ends at 3.33 Hz (F (44, 2) = 3187.8, p< 0.001), including many multiple regions (see Fig. 2.c.i and Table S2) and peaking in the left secondary auditory cortex (L-PBelt-ROI-lh). On In the right hemisphere, the cluster peaks ocipitaly at the V7 in occipital cortex, specifically in V7 (R-V7-ROI-rh), the significant di"erence is a bit slower: from 2 until 3.6 Hz and extends over slightly higher frequencies, from 2 to 3.6 Hz (F (44, 2) = 608.07, p < 0.01); See all regions in (See Fig.2.c.ii and for a list look at Table S1). all regions are shown in Fig. 2.c.ii and listed in Table S1.

Line 406: There, we demonstrated enhanced ocular tracking in late-deaf compared to hearing individuals so stronger visual involvement in late deafs (as the group is relying more on lip movements) might be increasing ocular tracking. Stronger visual involvement in late- deaf individuals, who rely more on lip movements as a compensatory strategy for reduced hearing abilities, may therefore contribute to increased ocular tracking. Line 447: Since sighted individuals integrate audiovisual information when listening to a speaker in everyday life, it could make sense that auditory input is sent to the eyes. Be- cause sighted individuals typically integrate audiovisual information during speech perception, auditory input may also be conveyed to the oculomotor system. Intriguingly, in this data, the mean syllable rate is at 6 Hz, so low-level auditory processing would happen in temporal regions for local computations and would drive the ocular tracking of speech. Intriguingly, in the present data the mean syllable rate is approximately 6 Hz, suggesting that low-level auditory processing in temporal regions may support local computations and, in turn, drive o1c6ular tracking of speech.

Revised Text (Proof reading over the whole manuscript) Line 456: This (→6 Hz) peak at syllable rate in sighted individuals could reflect the functional integration of the eyes into the auditory system. Importantly, this is not being explained by audiovisual listening experience, as if this was the case also the late blind participants would show increased tracking at 6 Hz, also sightedness seems to be required. While we find this e"ect of increased tracking at →6 Hz with coherence, using the mTRF approach, no statistically significant e"ect was found. This could be the case as the mTRF results rely on a single summary metric (prediction accuracy) and for coherence we have the frequency resolution. This (→6 Hz) peak at the syllable rate in sighted individuals may reflect functional involvement of the oculomotor system in auditory processing. Importantly, this e!ect is unlikely to be explained by audiovisual experience alone, as late-blind participants would be expected to show similarly increased tracking at 6 Hz if this were the case; instead, it appears to depend on visual experience. Although coherence reveals increased tracking at 6 Hz, the mTRF approach did not yield a statistically significant e!ect. This likely reflects methodological di!erences, as mTRF relies on a single summary metric (prediction accuracy), whereas coherence provides frequency-resolved information.

Line 473: Unlike neural tracking, ocular tracking was not significantly a"ected by the stimulus condition. That said, we were unable to prove the absence of an e"ect, as the data did not meet the criteria for statistical equivalence. Unlike neural tracking, ocular tracking was not significantly a!ected by the stimulus condition. That said, we were unable to prove the absence of an e!ect, as the data did not meet the criteria for statistical equivalence.

Line 480: However, previously a negative relationship between semantic comprehension and ocular speech tracking has been demonstrated (Schubert et al., 2024), which would not speak for the same e"ect in the eyes as neurally observed. However, previous work has reported a negative relationship between comprehension and ocular speech tracking (Schu- bert et al., 2024) - in other words, lower ocular tracking when comprehension is higher - which leads us to speculate that the eyes may not show stronger tracking with better comprehension, as is commonly reported in neural data. This could be the reason why we do not observe the typical vocoding e!ect in the ocular data.

Line 191 Further, we assume that the distinct spatial separation of the ocular sources allows them to be modeled as independent sources - probably even better than a single brain area being located less separated. We want to emphasize, that the distinct spatial location of the eyes allows them to be modeled as independent, likely even more reliably than a single brain area with less spatial separation.
