# Supplementary figures and images for: Ocular Speech Tracking Persists in Blindness, but Its Dynamics and Oculo-Cerebral Connectivity Depend on Visual Status
Source: eNeuro. 2026 Jul 7;13(7):ENEURO.0041-26.2026. doi: 10.1523/ENEURO.0041-26.2026 (PMC13349464; doi:10.1523/ENEURO.0041-26.2026)

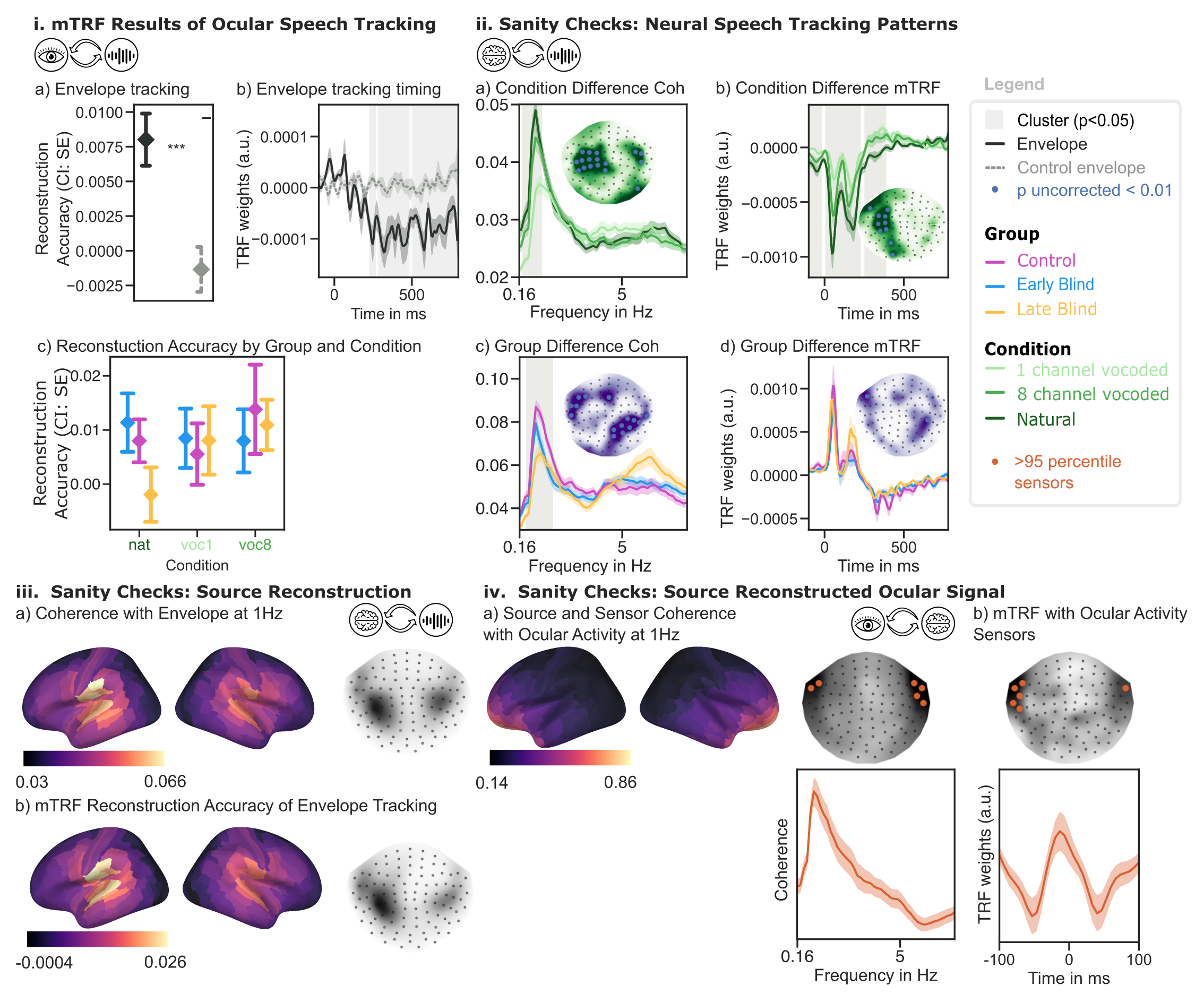

Supplement: Figure 1-1 — Ocular and neural speech tracking mTRF analyses and source reconstruction sanity checks. i. Ocular tracking mTRF results. a) Reconstruction accuracy (mean ± SE) of the mTRF model for the speech envelope, compared against a control envelope. Significant tracking is observed for the true envelope (***$p < 0.001$). b) Average temporal response function (TRF weights) across participants. Shaded areas indicate ± SE. Note that the sign of the mTRF weights is not interpretable, as the polarity of the underlying signals is arbitrary. c) Reconstruction accuracy across groups (sighted controls, early blind, late blind) and conditions. A mixed-design ANOVA with the dependent variable Ocular Tracking and factors Group (between-subjects) and Condition (within-subjects) revealed no significant main effect of group ($F(2, 44) = 0.27$, $p = .765$, $\eta^2_g = 0.006$), no significant main effect of condition ($F(44) = 0.73$, $p = .485$, $\eta^2_g = 0.009$), and no significant interaction between group and condition ($F(4, 88) = 0.93$, $p = .448$, $\eta^2_g = 0.022$). ii. To show maximal differences between groups and conditions, and to make sure that the ocular effects are not based on neural speech tracking effects, we plot the sensors with uncorrected $p < 0.01$ (blue) in a time or frequency resolution. Caution: Plots do not do not represent cluster-corrected statistical effect. a) Coherence between selected sensors and the speech envelope at 1 Hz. b) Condition differences in mTRF weights for the selected sensors. c) Group differences in coherence spectra. d) Group differences in mTRF weights. iii. Source reconstruction sanity checks. a) Source-level coherence with the speech envelope at 1 Hz. b) Source-level mTRF reconstruction accuracy. iv. Source reconstructed ocular signal sanity checks a) Coherence between reconstructed ocular activity and neural data at $\sim $1 Hz. This is plotted for both source reconstructed neural data and raw sensor data. The orange sensors reflect the [file eneuro-13-ENEURO.0041-26.2026-s006.tif]

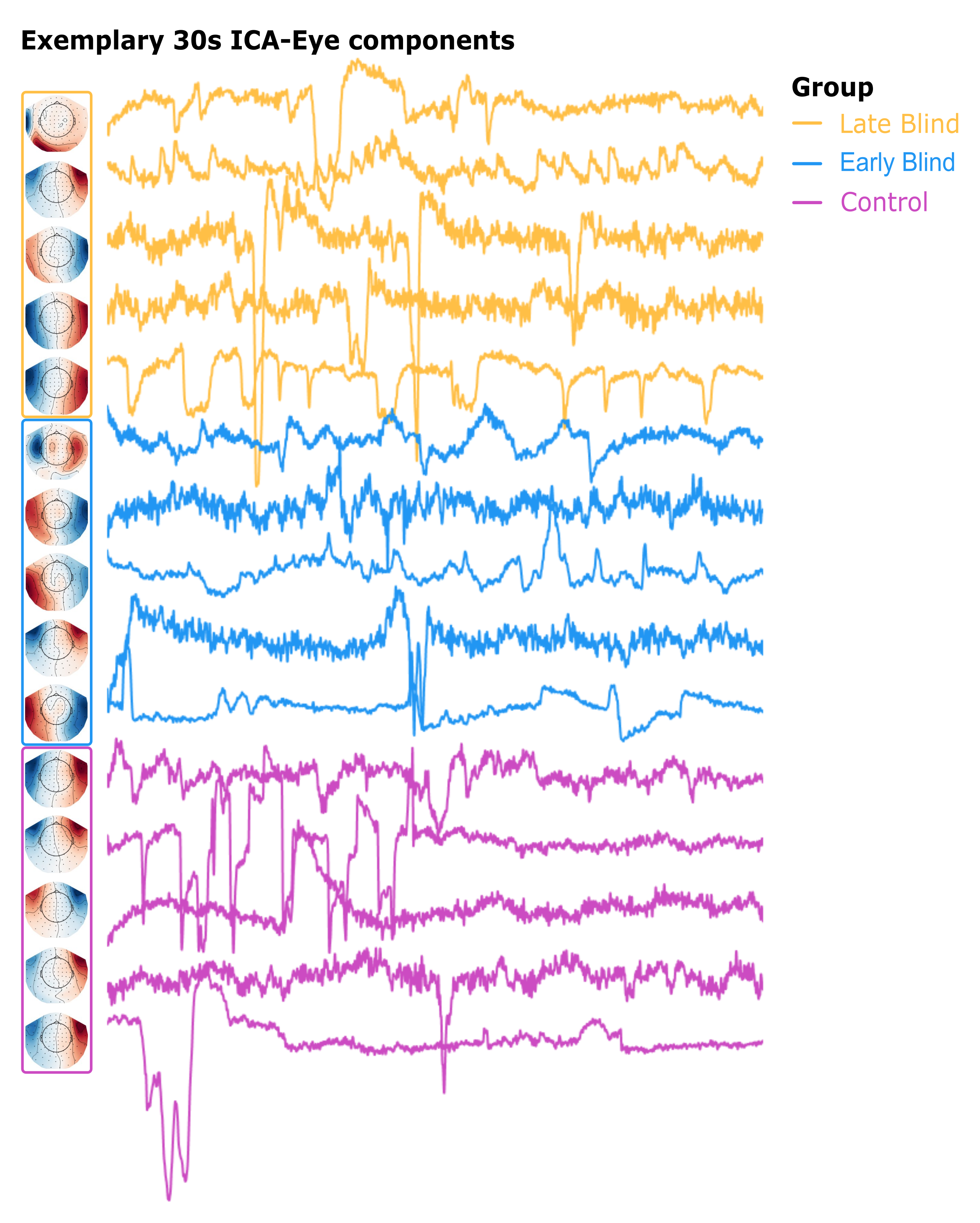

Supplement: Figure 1-2 — Representative ocular-related ICA components and corresponding time courses across groups. Example independent components classified as ocular-related are shown for five participants per group (early blind, late blind, sighted controls), together with 30-second segments of their time courses. Note that the between-subject variability within the blind groups was higher than in then sighted group, which was one reason for why we opted for source reconstruction. See Methods/MEG Data Preprocessing section for information on ICA-eye channel selection. Download Figure 1-2, TIF file. [file eneuro-13-ENEURO.0041-26.2026-s007.tif]

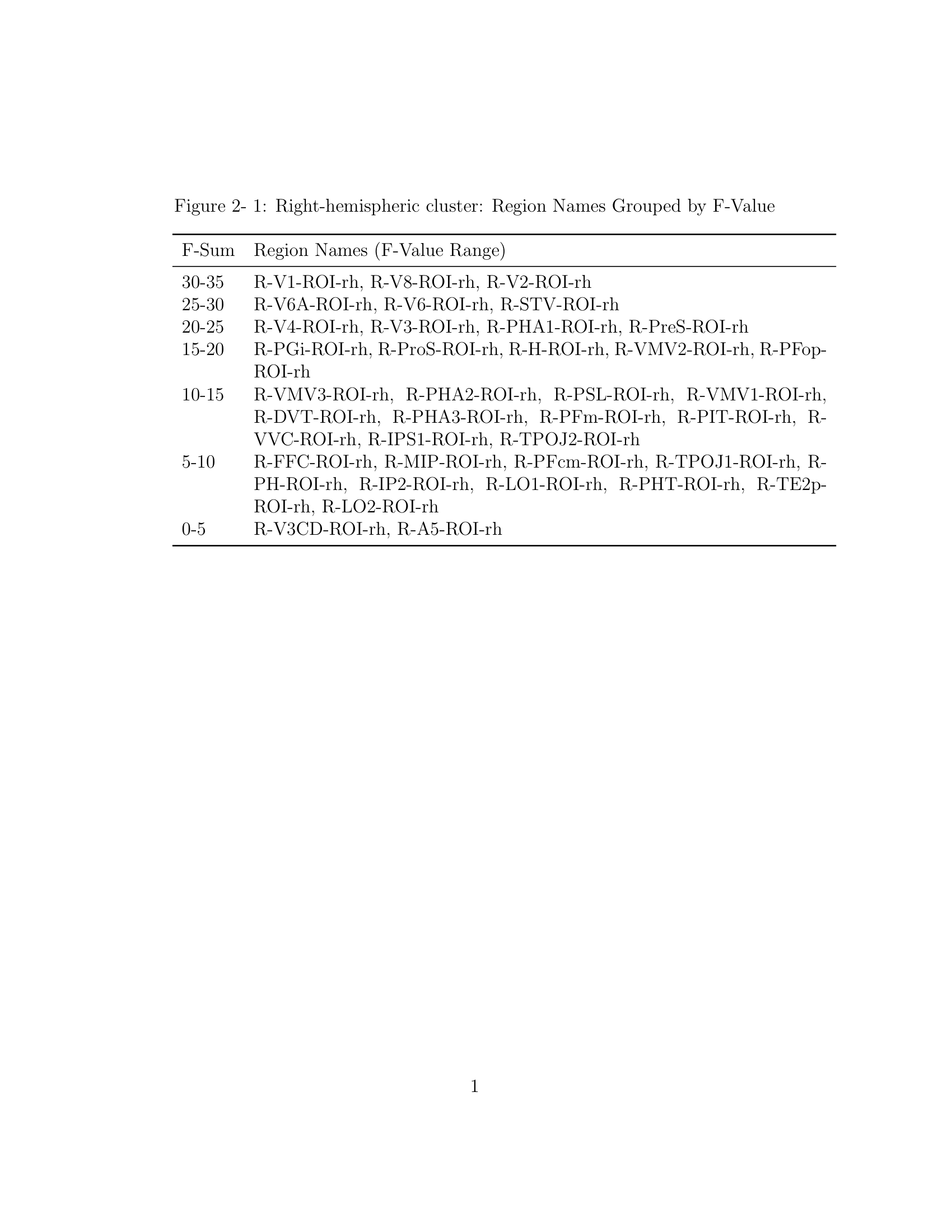

Supplement: Figure 2-1 — Group difference oculo-cerebro connectivity. In Fig.2.c.ii, we show the group difference of oculo-cerebro connectivity. Here we list the HCPMMP1 labels of the regions included in clusters in the right hemisphere. The regions are ordered by their contribution to the cluster statistics. The cluster in the right hemisphere spans frequencies from 2 to 3.66 Hz ($F(2,44)=608.07$, $p<0.01$) and peaks in occipital cortex, specifically in V7 (R-V7-ROI-rh). Download Figure 2-1, TIF file. [file eneuro-13-ENEURO.0041-26.2026-s008.tif]

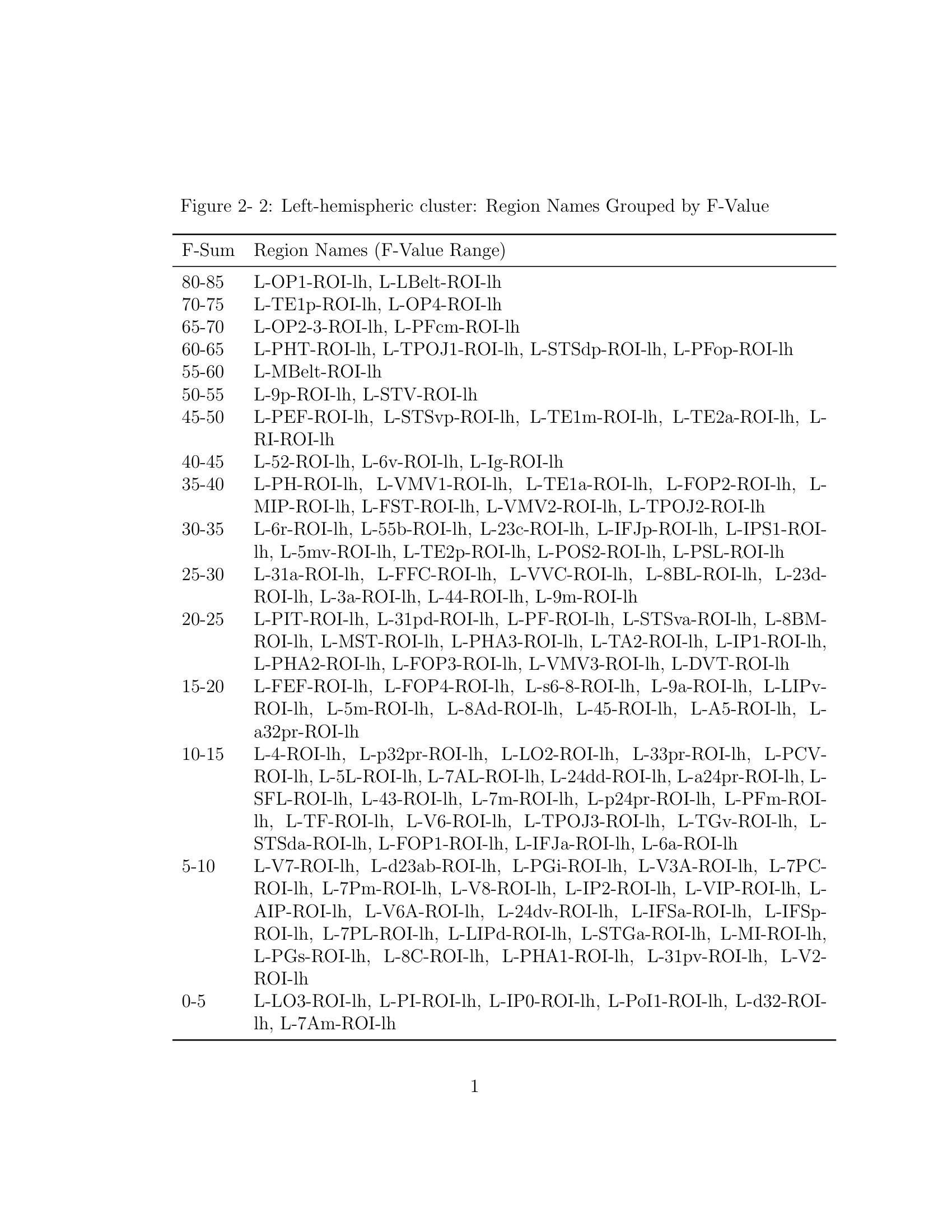

Supplement: Figure 2-2 — Group difference oculo-cerebro connectivity. In Fig.2.c.ii, we show the group difference of oculo-cerebro connectivity. Here we list the HCPMMP1 labels of the regions included in clusters in the left hemisphere. The regions are ordered by their contribution to the cluster statistics. The cluster in the left hemisphere spans frequencies from 0.17 to 3.33 Hz ($F(2, 44)=3187.8$, $p<0.001$) and peaks in the left secondary auditory cortex (L-PBelt-ROI-lh). Download Figure 2-2, TIF file. [file eneuro-13-ENEURO.0041-26.2026-s009.tif]
